# XOmiVAE-Inspired Transcriptomic Analysis of Chronic Pruritus of Unknown Origin: A Cutaneous State Distinct from Healthy Skin but Not Robustly Separable from Atopic Dermatitis

**DOI:** 10.3390/biomedicines14092000

**Published:** 2026-09-05

**Authors:** Yue-Min Zou, Man-Ning Wu, Dong-Mei Zhou, Wen-Bo Jiang, Yan-Ping Bai

**Affiliations:** 1 School of Traditional Chinese Medicine, Beijing University of Chinese Medicine, Beijing 100029, China; 2 Beijing Hospital of Traditional Chinese Medicine, Capital Medical University, Beijing 100010, China; 3 National Center for Integrative Medicine, China-Japan Friendship Hospital, Beijing 100029, China

**Keywords:** chronic pruritus of unknown origin, atopic dermatitis, prurigo nodularis, skin transcriptomics, variational autoencoder, interpretable machine learning, neuroimmune interaction, nested cross-validation, label permutation, signature stability

## Abstract

**Background/Objectives:** Chronic pruritus of unknown origin (CPUO) is mechanistically poorly resolved, and its relationship to atopic dermatitis (AD) is unclear. We asked how much apparent separation survives removal of leakage. **Methods:** Public bulk skin data (GSE237920; four healthy, four AD, four CPUO) were analyzed by differential expression and an XOmiVAE-inspired latent model, with public single-cell and atlas resources for context. Every label-dependent step was then re-executed inside each cross-validation fold, with label permutation and 1000 bootstraps. A frozen composite niche score was applied unchanged to prurigo nodularis (GSE210854) and AD (GSE174582) cohorts. **Results:** Fully nested, the latent classifier separated CPUO from healthy skin (AUC 1.000; permutation *p* = 0.020) but not from AD (0.741; *p* = 0.140); randomized labels also reached 1.00. The composite score reached 0.919 against healthy skin but did not exceed its null (*p* = 0.086). Six genes recurred in ≥80% of bootstraps, five within the signature. Cell-type-restricted blood analysis recovered CPUO-associated monocyte *CCL3* upregulation. In both validation cohorts, the frozen score was reduced, not elevated, in lesional skin. **Conclusions:** CPUO skin differs from healthy skin and occupies the low-inflammatory, epidermal-stress-dominated pole opposite lesional inflammatory skin, but cannot be robustly separated from AD at twelve samples.

## 1. Introduction

Chronic pruritus of unknown origin (CPUO) is a clinically challenging syndrome in which persistent itch occurs without an immediately identifiable dermatologic, systemic, or neurologic cause after routine evaluation [1,2]. The condition is burdensome for patients and difficult to treat, in part because the biological substrate of CPUO remains unclear [3]. Unlike inflammatory dermatoses with recognizable lesions and established immune pathways, CPUO may involve more subtle tissue programs that are not captured by conventional clinical classification: immune dysregulation has been documented in a subset of affected patients [4], lesion-free skin from patients with chronic itch of unknown origin can nonetheless show an enhanced TH2 immune profile [5], and circulating interleukin-31 has been proposed as a candidate mediator [6]. A better understanding of CPUO skin biology is therefore needed to distinguish disease-specific programs from nonspecific inflammation and from comparator disorders such as atopic dermatitis (AD). Two recent syntheses frame what is and is not established. A 2026 systematic review of 52 studies concludes that CPUO pathogenesis is multifactorial, implicating type 2 immune dysregulation, neurogenic mediators, metabolic alterations, and aging-related immune change, and that the treatment literature remains of low quality with few randomized controlled trials [7]; a contemporaneous therapeutic review reaches the same conclusion from the clinical side [8]. The work published since has characterized CPUO systemically rather than cutaneously: systemic immune profiling reports elevated serum levels of Mas-related G protein-coupled receptor X2 (MRGPRX2), a mast-cell-associated receptor, and identifies a distinct inflammatory signature [9], while plasma metabolomic profiling identifies a circulating biomarker signature that distinguishes CPUO from healthy controls [10]. A corresponding description of the skin itself is still missing, and that is the gap this study addresses.

Most molecular studies of chronic itch have focused on inflammatory cytokines, barrier dysfunction, neural sensitization, or individual candidate genes. These approaches have been informative but may miss coordinated multicellular programs in the skin. Itch arises from communication among keratinocytes, immune cells, stromal cells, vascular compartments, and peripheral nerves [11]; sensory neurons can co-opt classical immune signaling pathways to sustain chronic itch [12], and epithelium-derived alarmins such as IL-33 act directly on sensory neurons [13]. Recent syntheses of the neuroimmune axis in itch go further, treating sensory neurons and immune cells as one integrated system rather than two adjacent ones, and extending the circuit from peripheral neuron-immune signaling to central glial contributions [14,15]; experimental work now shows that type 2 cytokines remodel the architecture of cutaneous sensory nerves and not only their excitability [16]. These interactions may be organized within local tissue neighborhoods rather than reflected by a single circulating biomarker. The lack of true CPUO skin single-cell and spatial datasets has limited direct interrogation of this tissue organization.

Recent single-cell and spatial studies in skin and other barrier tissues have emphasized multicellular neighborhoods as units of tissue state; in psoriatic disease, spatial transcriptomics stratified severity through emergent cellular ecosystems [17]. Fibroblast-macrophage equilibrium has been shown to govern skin integrity in mouse genetic models [18], and reciprocal fibroblast-macrophage interactions are increasingly recognized as regulators of tissue state in health, fibrosis, and cancer [19]. These concepts are relevant to CPUO but should be applied cautiously: mouse genetic evidence and reference atlases derived from other diseases or developmental stages cannot replace disease-specific human tissue measurements. They can, however, provide a framework for contextualizing bulk transcriptomic signatures and generating spatial or cellular hypotheses.

Conventional differential expression and pathway analysis tests genes one at a time and models each contrast as a linear effect, so coordinated programs distributed across many individually weak changes can be missed; with 20,595 genes measured in 12 samples, the number of variables exceeds the number of observations by three orders of magnitude. Latent-variable models are a complementary approach, compressing the measured genes into a small number of axes that can absorb non-linear structure and making those axes, rather than individual genes, the unit of interpretation. Interpretable deep learning offers another route to extract disease-associated patterns from high-dimensional transcriptomic data. XOmiVAE was developed for multi-omics cancer classification and couples a variational autoencoder with a supervised head and native DeepSHAP-based explanation [20]. Its architecture is conceptually suited to identifying latent representations and connecting them to interpretable molecular features. We selected it on that architectural rationale rather than because it is established for cohorts of this size, and we note that the model is not unsupervised: a classification head is trained on the group labels, and the consequences of that dependence for the validity of the reported performance are quantified in Section 3.9. In small cohorts, such models must be used carefully: predictive metrics are prone to overfitting, and biological interpretation should be prioritized over claims of clinical classification. In this study, we therefore used an XOmiVAE-inspired, expression-only adaptation—which retains the variational encoder/decoder and supervised head but replaces the native DeepSHAP module with reproducible post hoc contribution metrics (Section 2)—as an exploratory, interpretable latent-state model rather than as a validated diagnostic tool.

In this study, we integrated public bulk skin transcriptomes from CPUO, AD, and healthy controls (GSE237920) with peripheral blood mononuclear cell (PBMC) scRNA-seq data from CPUO and healthy cohorts (GSE295457), alongside a public skin spatial transcriptomic reference (GSE202011) and a public human dorsal root ganglion (DRG)/sensory neuron atlas (GSE245310). This integration aimed to test the hypothesis that CPUO is associated with a distinct neuroimmune-stromal cutaneous latent state. We first characterized the bulk transcriptomic landscape of CPUO, then applied the latent model to identify transcriptional programs and CPUO-driving genes. We next evaluated whether these skin-derived signatures were detectable within circulating immune compartments, both in aggregate and within individual immune cell types, and projected them onto the public skin spatial and DRG references for atlas-guided inference. Because those references derive from psoriatic skin and from embryonic DRG, respectively, they provide anatomical and cellular context rather than disease-matched validation. This study is designed as a hypothesis-generating computational analysis rather than a causal or clinical diagnostic validation. Because the discovery cohort contains twelve samples, we additionally treated the validity of our own procedure as an object of study: every label-dependent step was re-executed inside each cross-validation fold, the complete nested pipeline was repeated under randomized labels, and the stability of the resulting gene signature was quantified by resampling. Upstream feature selection is a recognized source of optimistic bias in small transcriptomic cohorts [21,22], and these analyses are therefore reported alongside the biological results rather than in an appendix, because they determine how much of the biology can be claimed.

## 2. Materials and Methods

### 2.1. Study Design and Multi-Modal Data Integration

In this study, we integrated public bulk skin transcriptomic data (GSE237920) and peripheral blood mononuclear cell (PBMC) scRNA-seq data from CPUO and healthy cohorts (GSE295457), alongside a public skin spatial transcriptomic reference (GSE202011) and a public human dorsal root ganglion (DRG)/sensory neuron atlas (GSE245310). This multi-modal approach aimed to generate a hypothesis-driven computational model of CPUO. Direct transcriptomic evidence for CPUO skin was derived exclusively from the GSE237920 bulk dataset. The GSE295457 dataset was used to profile the peripheral immune compartment and was not intended as a surrogate for cutaneous single-cell validation. The spatial and DRG atlases were applied strictly for atlas-guided inference and biological interpretation; readers should note that GSE202011 is a psoriatic-disease spatial resource and GSE245310 profiles human embryonic DRG (gestational weeks 7–21), so neither is disease- nor age-matched to CPUO.

No wet-lab experimental validation was conducted in this study.

### 2.2. Data Acquisition and Preprocessing

Bulk skin transcriptomic data were retrieved from the Gene Expression Omnibus (GEO; accession GSE237920 [23]) using the GEOquery R package. Phenotypic metadata were curated to classify samples into healthy controls (HC), atopic dermatitis (AD), and CPUO groups. Probe identifiers were mapped to gene symbols using platform annotations; probes mapping to ambiguous symbols were removed, and duplicated symbols were collapsed by mean expression. Missing values were imputed via gene-level medians. Expression values were log2-transformed where required, and lowly expressed genes (expression ≤ 1 in >75% of samples) were filtered out. Batch structure was assessed in two ways. The curated metadata contained no usable batch variable with more than one level, so ComBat correction could not be applied and was skipped; independently, surrogate variable analysis (SVA) on the normalized matrix did not identify latent structure requiring correction. The normalized matrix was therefore used unchanged for all downstream analyses.

### 2.3. Differential Expression and Functional Enrichment Analysis

Differential expression analysis was performed using the limma package. Linear models were fitted for CPUO vs. HC, AD vs. HC, and CPUO vs. AD contrasts, with empirical Bayes moderation applied to compute contrast statistics. Significant differentially expressed genes (DEGs) were defined by a Benjamini–Hochberg-adjusted *p*-value < 0.05 and an absolute log2 fold change > 0.5. Functional enrichment of significant DEGs—encompassing Gene Ontology (GO), Kyoto Encyclopedia of Genes and Genomes (KEGG), and Reactome pathways—was performed using clusterProfiler and ReactomePA. Gene Set Enrichment Analysis (GSEA) was executed utilizing genes ranked by moderated t-statistics. Pathway activity scores for predefined and custom functional modules (e.g., neuroactive ligand-receptor interaction, keratinocyte differentiation, fibroblast activation) were computed using Gene Set Variation Analysis (GSVA). Genes significant in CPUO versus HC but not significant in AD versus HC are referred to throughout as CPUO-versus-HC-enriched rather than CPUO-specific. A difference in significance between two contrasts is not evidence of a significant difference between them [24]; the direct CPUO-versus-AD contrast is reported separately and is not used to justify the earlier term. The complete gene membership of every custom pathway module scored by GSVA is listed in Supplementary Table S6.

### 2.4. Latent Modeling and Feature Attribution

An XOmiVAE-inspired, expression-only adaptation was used to identify an interpretable CPUO-associated latent transcriptomic state. To avoid overstating equivalence with the published method [20], we specify the relationship explicitly. Retained from the original XOmiVAE: the variational autoencoder architecture with a supervised classification head on the latent representation, the use of latent dimensions as interpretable disease-state axes, and the principle of connecting latent structure back to individual genes. Replaced: the original DeepSHAP-based attribution, which is the defining feature of XOmiVAE, could not be computed for this adapter implementation and was substituted with a deterministic post hoc contribution score (below). Omitted: the multi-omics input branches, since only expression data were available. The rationale for these substitutions was reproducibility under a 12-sample cohort, where gradient-based attribution on a deep model is unstable; the cost is that the resulting gene rankings are not DeepSHAP values and should not be compared to published XOmiVAE attributions. The model used 2000 input genes, a 4-dimensional latent space, a single hidden layer, and a linear classification head on the latent representation; it was trained for 450 epochs with the Adam optimizer at a learning rate of 1 × 10^−3^ under a fixed random seed (237920) in PyTorch. Robustness was evaluated via leave-one-out cross-validation (LOOCV); input-gene selection was recomputed inside each fold from training samples only, although the selection rule prioritized a CPUO-versus-HC-enriched and CPUO-versus-AD DEG list derived from all 12 samples, so the procedure is nested with respect to variance-based selection but only partially nested with respect to that prior. Gene-level contributions were calculated by integrating projected encoder-to-latent weights, observed gene-latent correlations, and standardized CPUO expression effect sizes, then combined with DEG and pathway evidence to define the primary CPUO-driving signature. For independent evaluation without reference to the code repository, the implementation is specified in full. The encoder comprised a single fully connected hidden layer of min(64, max(8, p/8)) units for p input genes—sixty-four units at both the 2000-gene and 700-gene budgets—with rectified linear unit (ReLU) activation and dropout of 0.05 applied during training only, followed by two linear maps to the 4-dimensional posterior mean and log-variance; the log-variance was clipped to [−8, 8] and the latent sample drawn as z = μ + ε·exp(0.5·logvar) with ε ~ N(0, 1). The decoder mirrored this structure (linear, ReLU, linear), and the supervised head was a single linear layer applied to the posterior mean rather than to the sampled latent. The training objective was the sum of a mean-squared-error reconstruction term, a Kullback–Leibler divergence term weighted by β = 0.02, and a class-weighted cross-entropy term weighted by 0.35. Optimization used Adam (learning rate 1 × 10^−3^, weight decay 1 × 10^−4^) for up to 450 epochs with early stopping after 80 epochs without improvement, and weights were initialized by the PyTorch default uniform scheme. The primary run used seed 237920; the resampling analyses in Section 2.11 varied the seed across repeats and are reported as mean ± standard deviation, so no result depends on a single random initialization. To make the several thousand end-to-end refits required by those analyses computationally feasible, the limma contrast, the model, and the scoring chain were re-implemented in NumPy; this port was verified by finite-difference gradient checking (maximum relative error 5.1 × 10^−8^) and by exact reproduction of the differential expression counts and gene identities obtained in R.

### 2.5. Module Assignment

Signature genes were assigned to one of four modules—neuro-sensory, myeloid-immune, stromal-vascular, and keratinocyte-stress—using a two-stage rule. In the first stage, a gene was assigned by symbol-prefix matching against curated keyword lists for each module (neuro-sensory: *NGF*, *NTRK*, *BDNF*, *TAC*, *CALC*, *TRPV*, *TRPA*, *SCN*, *HTR*, *OPRM*, *NPPB*, *OSMR*, *IL31*, *NRP*, *SEMA*, *PLXN*, *ROBO*, *SLIT*, *EPH*; *myeloid-immune*: *IL*, *CCL*, *CXCL*, *CCR*, *CXCR*, *LST1*, *S100*, *FCGR*, *CD14*, *CD68*, *TNF*, *NLRP*, *CSF*, *IFN*, *CD3*, *CD4*, *CD8*, *HLA*; stromal-vascular: *COL*, *FN1*, *LAMA*, *LAMB*, *ITGA*, *ITGB*, *MMP*, *PECAM*, *VWF*, *KDR*, *PDGFR*, *ACTA2*, *TAGLN*, *VCAM*, *ICAM*, *SELE*, *RGS5*, *MCAM*, *THBS*, *SPP1*; keratinocyte-stress: *KRT*, *FLG*, *LOR*, *IVL*, *SPRR*, *DSG*, *DSC*, *TGM*, *S100A*, *AQP3*, *CLDN1*, *TP63*, *BHLHE*, *TXNRD*, *HMOX*, *NQO*, *GPX*, *PRDX*). In the second stage, genes not matched by any keyword were assigned to the module whose mean z-scored module expression they correlated with most strongly in absolute value. This rule is heuristic, and its limits should be recognized. Only 20 of the 201 signature genes were assigned by keyword; the remaining 181 were assigned by correlation, which reflects co-expression in this cohort rather than curated function. The keyword lists are also prefix-based and therefore capture families rather than individual functions: *NRP1* and *NRP2* match the neuro-sensory ‘*NRP*’ pattern because neuropilins are semaphorin co-receptors on sensory axons, but both are equally well established as VEGF co-receptors on vascular endothelium, and their placement in the neuro-sensory rather than the stromal-vascular module should be treated as a labeling convention rather than as a functional claim. Module assignment, assignment basis (keyword versus correlation), and assignment confidence are reported for every gene in Supplementary Table S2. Because this assignment is derived within the same twelve samples that define the signature, its stability was quantified by resampling (Section 2.11), and the result is reported in Section 3.9. The term “module” is retained for continuity with the figures, but it should be read as a keyword- and co-expression-annotated gene group rather than as an independently established transcriptional program. Because the neuro-sensory module contains only 13 genes and *NRP1* and *NRP2* are two of them, we repeated every module-level and composite analysis with both genes reassigned to the stromal-vascular module as a pre-specified sensitivity analysis; the result is reported in Section 3.8.

### 2.6. SHAP Sensitivity Analysis

Tensor-level DeepSHAP attribution could not be computed for the adapted model itself. To provide some external check on the gene rankings, SHAP values were instead computed for three surrogate classifiers—logistic regression, random forest, and linear support vector machine—trained on the same input matrix and class labels, and the top 10, 20, 50, and 100 SHAP-ranked genes from each were compared with the 201-gene signature. This is a comparison against different model families, not an attribution of the adapted model, and it was pre-specified as a sensitivity analysis whose result would be reported irrespective of direction.

### 2.7. PBMC scRNA-Seq Processing and Pseudo-Bulk Analysis

The public GSE295457 [25] single-cell dataset was processed to profile the PBMC landscape using Scanpy-compatible pipelines. Raw data (10X Genomics format) and SOFT-formatted metadata were retrieved from GEO to assign sample-level diagnostic labels (CPUO and HC); this dataset lacked AD controls. For group-aware differential analysis, QC-retained cells were aggregated into sample-level pseudo-bulk profiles. Following duplicate-gene resolution, library sizes were converted to counts per million (CPM), and log2(CPM+1) values were used for group comparisons. Differential testing was restricted to genes with CPM > 1 in at least two samples and ≥10 total counts across all pseudo-bulk profiles. CPUO vs. HC comparisons used Welch’s *t*-test with GEO samples as biological replicates (*n* = 4 per group), followed by Benjamini–Hochberg correction, with significance defined as FDR < 0.05 and |log2 fold change| > 0.5. Cell-type composition was assessed using sample-level fractions via the Mann–Whitney U test with FDR correction. Because whole-cell aggregation averages across all circulating populations and can mask compartment-restricted signals, the same comparison was additionally performed within individual cell types. Sample-by-cell-type pseudo-bulk profiles were reconstructed directly from the raw 10X count matrices, restricted to the QC-retained barcodes and using the cell-type labels assigned in the main annotation, and analyzed with the identical CPM normalization, expression filter, Welch’s *t*-test and Benjamini–Hochberg procedure applied independently within each compartment (classical monocytes, non-classical monocytes, monocytes combined, NK cells, T cells combined, and dendritic cells). Given the cohort size, all pseudo-bulk analyses—whole-cell and cell-type-restricted—are interpreted as exploratory.

### 2.8. Spatial and DRG Reference Atlas Mapping

For spatial contextualization, signature modules were mapped onto a public skin spatial transcriptomic reference (GSE202011), a psoriatic-disease resource comprising matched lesional and non-lesional psoriatic samples and healthy controls [17]. Module scores were calculated as the mean log1p-normalized expression across signature genes and summarized by spatial neighborhood annotation. For neuroimmune interpretation, a public human DRG atlas (GSE245310) was used; this is a single-nucleus dataset of human embryonic DRG spanning gestational weeks 7–21 [26], and original cell-type labels were mapped to standardized categories before scoring. Signature modules and canonical itch-related genes (e.g., *SCN10A*, *NTRK1*, *IL31RA*) were scored across standardized neural and glial populations as the fraction of cells with non-zero normalized expression and the mean normalized expression. Putative skin-to-DRG ligand-receptor axes were systematically queried, including *CCL3-CCR1*, CXCL12*-CXCR4*, *NGF-NTRK1*, *BDNF-NTRK2*, *IL31-IL31RA*, *OSM-OSMR*, *CCL2-CCR2*, and *TGFB1-TGFBR1*/*TGFBR2*. Neither atlas is disease- or age-matched to CPUO, and all resulting statements are atlas-guided inference rather than validation.

### 2.9. Fibroblast-Myeloid Dominance and CPUO Niche Scoring

Stromal-immune balance was inferred from a curated 16-gene fibroblast marker set and a curated 16-gene myeloid marker set scored on the bulk expression matrix; both lists are given in full in Supplementary Table S7. A fibroblast-myeloid dominance score was defined as z(fibroblast marker score) minus z(myeloid marker score); positive values indicate relative fibroblast predominance. Two scalings of the underlying marker scores appear in this paper, and we distinguish them explicitly, because they are not interchangeable. The descriptive group comparisons in Section 3.6 score each marker set as the z-transformed mean expression of its genes. The dominance score that enters the composite niche score, and that is frozen and re-applied to the validation cohorts in Section 2.10, scores each marker set as the mean within-sample percentile rank of its genes, because only the rank version is invariant to platform-specific intensity scaling and can therefore be transported across microarray platforms. Every composite-score result in Section 3.8 and Section 3.10, and every component-level value reported in Section 3.8, uses the rank version. The composite CPUO neuroimmune-stromal niche score is the mean of five z-scored, sign-aligned components: the neuro-sensory, myeloid-immune, stromal-vascular, and keratinocyte-stress module scores plus the fibroblast-myeloid dominance score. Two of those five components carry myeloid information and are both oriented against it: the myeloid-immune module score, and the myeloid half of the dominance score. The effective weight given to the myeloid program is therefore approximately two-fifths rather than one-fifth, and we accordingly report throughout a four-component variant that omits the dominance score. That four-component variant is also what the nested re-validation of Section 2.11 evaluates. Sign alignment used the group labels of the same samples in which the score was evaluated, so its contribution to apparent performance was quantified in two ways. First, leave-one-out evaluation was repeated with both z-scoring and sign alignment re-derived from the 11 training samples in each fold, so that the held-out sample contributed to neither. Second, a label-permutation test shuffled group labels 5000 times and repeated the entire z-scoring, sign-alignment, and scoring procedure within each permutation, yielding an empirical null distribution for the CPUO-versus-rest AUC. Exploratory receiver operating characteristic analyses are reported in the Supplementary Material only.

### 2.10. External Evaluation in Independent Public Skin Cohorts

Two independent public skin transcriptomic series were used to test whether the composite niche score behaves as a CPUO-associated quantity or as a generic marker of inflamed or pruritic skin. GSE210854 [27] provides Affymetrix Human Gene 1.0 ST (GPL6244) profiles of pruritic lesional skin, paired non-pruritic non-lesional skin from the same five patients with prurigo nodularis (PN), and five healthy skin samples. GSE174582 [28] provides Affymetrix HG-U133A (GPL96) profiles of 4 normal, 7 uninvolved, and 23 involved atopic dermatitis skin samples (8 acute, 9 subacute, and 6 chronic lesions). Processed values were read from the GEO SOFT family files, probes were mapped to gene symbols using the corresponding platform annotation, and multiple probes per symbol were collapsed by retaining the probe with the highest mean signal. Everything learned from the discovery cohort was frozen: the 201-gene signature, its four module assignments, the curated 16-gene fibroblast and 16-gene myeloid marker sets, and the five component orientation multipliers (+1 neuro-sensory, −1 myeloid-immune, −1 stromal-vascular, +1 keratinocyte-stress, and +1 fibroblast-myeloid dominance). Module scores were computed exactly as in the discovery cohort, as the mean within-sample percentile rank of the module genes present, which makes them invariant to platform-specific intensity scaling. The fibroblast-myeloid dominance score and the z-scaling of each component were necessarily computed within each validation cohort, because cross-platform values are not directly comparable; no other parameter was re-estimated, and no orientation was re-derived. Group contrasts used exact Mann–Whitney U tests reported with Cliff’s delta and AUC; the within-patient PN contrast used an exact Wilcoxon signed-rank test, and a 10000-fold label permutation of the complete scoring procedure provided an empirical null distribution for the PN cohort. Three sensitivity analyses were pre-specified for this external evaluation and are reported in Section 3.10. First, the composite was recomputed without the fibroblast-myeloid dominance component, for the double-counting reason given in Section 2.9. Second, because the proportion of lesional samples differs markedly between the two series (5 of 15 in GSE210854 versus 23 of 34 in GSE174582), within-cohort z-scaling was replaced by z-scaling referenced to the healthy or normal group of each cohort alone, so that the scaling cannot be driven by the composition of the diseased samples. Third, because GSE174582 carries only 114 of the 201 signature genes, the discovery-cohort analysis was repeated after restricting every module to the genes measurable on that platform, to test whether the direction reversal is an artifact of coverage.

### 2.11. Nested Re-Validation, Label Permutation and Signature Stability

Three steps in the primary pipeline used the labels of all twelve samples before cross-validation. The differential expression contrasts were computed on the full cohort and written to disk; the fold-internal feature selection then read those lists, so that 480 of the 700 features selected in each leave-one-out fold were chosen using a list to which the held-out sample had contributed; and the orientation of each niche-score component was fixed from the CPUO and non-CPUO means of all twelve samples. Such upstream selection is a recognized source of optimistic bias in small transcriptomic cohorts [21,22,29,30]. We therefore re-executed the entire analysis under a fully nested protocol in which differential expression, feature selection, model training, driving-gene derivation, module assignment, z-scaling, and component orientation were all recomputed inside each fold from the training samples alone, with nothing else changed. The non-nested protocol was retained as a comparator so that the two differ only in where label information enters. Both were run at feature budgets of 700 and 2000 genes, over 20 and 10 repeats respectively, with a different random seed in each repeat. The nested re-validation evaluates the four-component variant of the composite score defined in Section 2.9; the five-component version reported in Section 3.8 is not re-evaluated under this protocol, so the two are different quantities, and the comparison between them changes both the protocol and the component set. Section 3.10 reports the discovery-cohort contrasts for both variants, which are identical.

To establish what the nested protocol produces in the absence of real structure, the group labels of the twelve samples were randomly permuted 300 times, preserving the 4/4/4 design, and the complete pipeline—differential expression onward—was re-run within each permutation, giving an empirical null distribution for every reported metric. Permutations were drawn from the 12!/(4!)^3^ = 34,650 distinct label assignments with duplicates rejected, so no assignment contributes twice, and the observed labeling is excluded; the resolution of the test is therefore set by the number of permutations drawn and not by the size of the assignment space. Empirical *p*-values are (1 + k)/(N + 1), where k is the number of permutations reaching or exceeding the observed value. At N = 300, this estimate is coarse: an empirical *p* of 0.020 corresponds to k = 5 and carries a Monte Carlo 95% interval of approximately 0.007 to 0.046, which we state rather than correct. The same procedure was applied to the non-nested protocol as a control, over 200 permutations, so that the effect of leakage on the observed statistic could be separated from its effect on the null.

Signature stability was assessed by re-deriving the complete driving-gene chain within 1000 stratified bootstrap resamples and within all twelve jackknife subsets, recording for each resample the size of the resulting signature, its Jaccard overlap with the reported 201-gene set, the module sizes, and the number of differentially expressed genes at each step; a gene was regarded as belonging to the stable core if it was selected in at least 80% of bootstrap resamples [29]. Finally, 95% confidence intervals for Cliff’s delta were obtained by exhaustive stratified bootstrap: with four samples per group, the resampling space contains 4^4^ × 4^4^ = 65,536 distinct configurations, all of which were enumerated, so the intervals carry no Monte Carlo error.

### 2.12. Statistical Analysis

Group comparisons for pathway activities and module scores used non-parametric tests (exact Mann–Whitney U for pairwise contrasts, Kruskal–Wallis for three-group contrasts). Because the cohort contains 4 samples per group, these tests have hard lower bounds on the *p*-values they can return: the smallest attainable two-sided *p* is 0.0286 for a 4-versus-4 Mann–Whitney test and 0.0073 for a 4/4/4 Kruskal–Wallis test. *p*-values at those bounds indicate complete rank separation and convey no further information about effect magnitude. Effect sizes are therefore reported as Cliff’s delta for all niche-score and module-level comparisons, and classification performance is supported by label-permutation testing rather than by ROC statistics alone. Kruskal–Wallis tests across the five niche-score components were corrected by the Benjamini–Hochberg procedure, and both raw and adjusted values are given. Benjamini–Hochberg correction was also applied to all genome-wide differential expression and enrichment analyses. Analyses were conducted in R 4.4.1 (limma 3.60.4, clusterProfiler 4.12.0, ReactomePA 1.48.0, GSVA 1.52.3, sva 3.52.0) and Python 3.12.13 (PyTorch 2.4.0, scanpy 1.10.2, scikit-learn 1.5.1, shap 0.46.0, numpy 2.0, pandas 2.2). Dataset accession numbers and analysis code are provided in the Data Availability and Code Availability statements. Cliff’s delta is reported with the exhaustive stratified bootstrap interval defined in Section 2.11 [31]; where CPUO is completely rank-separated from a comparator, that interval is degenerate at the boundary, for the structural reason given in Section 3.8. Where a Kruskal–Wallis test is reported for the twelve-sample cohort, the asymptotic chi-square *p*-value is given, and the exact *p* obtained by enumerating all 34,650 possible 4/4/4 label assignments is given alongside it wherever the two differ materially.

## 3. Results

### 3.1. CPUO Shows Extensive Transcriptomic Changes Versus Healthy Skin but Limited DEG-Level Separation from AD

**Figure 1:**
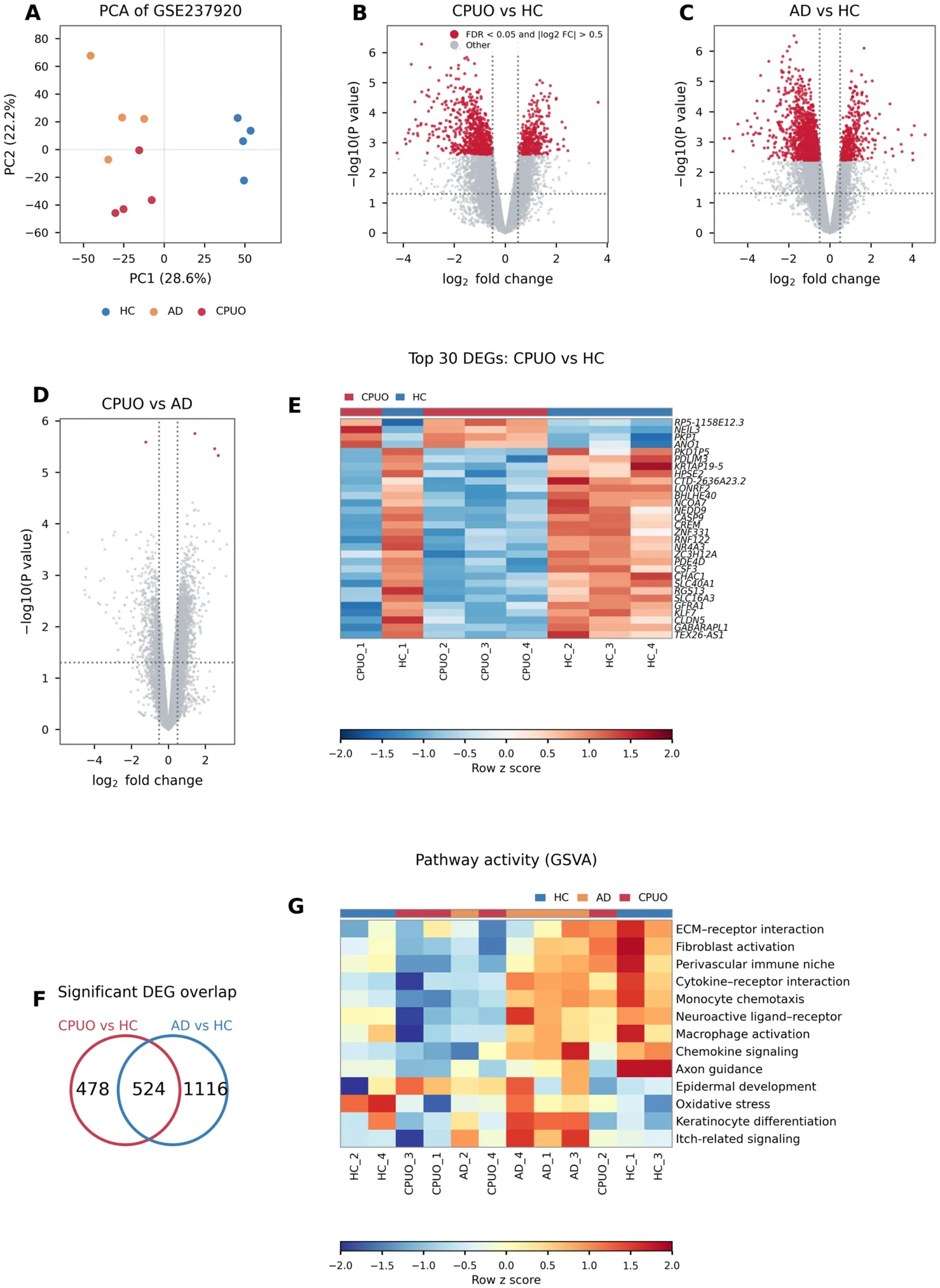
CPUO skin bulk transcriptomic landscape and differential expression. Bulk skin transcriptomes from GSE237920 were analyzed across healthy controls (HC; *n* = 4), patients with atopic dermatitis (AD; *n* = 4), and patients with chronic pruritus of unknown origin (CPUO; *n* = 4). (**A**) Principal component analysis of the 12 bulk skin samples; each point represents one sample and colors indicate the diagnostic groups. (**B**) Volcano plot for CPUO versus HC, identifying 1002 significant differentially expressed genes (DEGs). (**C**) Volcano plot for AD versus HC, identifying 1640 significant DEGs. (**D**) Volcano plot for CPUO versus AD, identifying four significant DEGs. In panels (**B**–**D**), red points satisfy an adjusted *p*-value < 0.05 and |log2 fold change| > 0.5; the grey dotted vertical lines indicate log2 fold-change thresholds of −0.5 and 0.5, and the grey dotted horizontal line indicates a nominal *p*-value threshold of 0.05. (**E**) Heatmap of the 30 most strongly ranked DEGs in the CPUO-versus-HC comparison, ordered by nominal *p*-value and displayed as row-wise z-standardized expression across the CPUO and HC samples. The upper annotation strip indicates sample group. (**F**) Venn diagram showing 478 CPUO-versus-HC-specific DEGs, 524 DEGs shared between the CPUO-versus-HC and AD-versus-HC comparisons, and 1116 AD-versus-HC-specific DEGs. (**G**) Hierarchically clustered heatmap of row-wise z-standardized gene set variation analysis (GSVA) pathway-activity scores across all 12 samples; the upper annotation strip indicates sample group. In panels E and G, blue denotes lower and red denotes higher row-wise standardized values, as shown by the color-scale legends.

The GSE237920 bulk skin transcriptomic cohort contained 12 samples, including four HC, four AD, and four CPUO samples after metadata harmonization and sample matching. The public metadata for this series report only diagnosis, tissue, and platform; age, sex, disease duration, itch severity, treatment history, and biopsy site are not deposited, and the available and unavailable fields for every sample are listed in Supplementary Table S1. After gene-symbol mapping, duplicate-gene handling, missing-value checks, log-scale assessment, and low-expression filtering, 20,595 genes were retained for downstream differential expression analysis. Batch structure was evaluated in two complementary ways. First, the curated GEO metadata contained no usable batch variable with more than one level, so explicit ComBat correction could not be applied. Second, surrogate variable analysis on the normalized matrix did not identify latent structure requiring correction. The normalized matrix was therefore carried forward unchanged and used as the ComBat-compatible input for downstream modules (Figure 1; Supplementary Figure S1).

Using limma with an adjusted *p*-value threshold of <0.05 and an absolute log2 fold-change threshold of >0.5, the comparison between CPUO vs. HC identified 1002 significant differentially expressed genes among 20,595 tested genes. The AD vs. HC contrast identified 1640 significant genes, whereas the comparison between CPUO and AD yielded only four significant discriminatory genes. CPUO-focused set analysis identified 478 CPUO-versus-HC-enriched DEGs, defined as significant in CPUO vs. HC but not significant in AD vs. HC, and 524 DEGs shared between CPUO vs. HC and AD vs. HC. The four CPUO vs. AD discriminatory genes were *SCCPDH*, *BHLHE40*, *PSG4*, and *PHF2P2*. Two of these warrant explicit caution: *PHF2P2* is an annotated pseudogene, and *SCCPDH* (saccharopine dehydrogenase-like oxidoreductase) has no established role in cutaneous or sensory biology; in small bulk cohorts, high ranking of pseudogenes and poorly characterized loci frequently reflects probe cross-hybridization or multi-mapping artifacts rather than biology, and neither gene is discussed further as a mechanistic candidate. Taken together, the near-absence of DEG-level differences indicates that conventional differential expression is underpowered to separate CPUO from AD in a cohort of this size; any claim that CPUO is distinguishable from AD therefore rests on the latent-state and composite-score analyses presented below rather than on this contrast (Figure 1). We no longer describe this set as CPUO-specific: a difference in significance between two contrasts is not evidence of a significant difference between them [24], and the direct CPUO-versus-AD contrast yielded only four genes. As shown in Section 3.9, the latent-state and composite-score analyses do not support a claim of distinctness from AD once label leakage is removed, and we therefore do not make it.

Functional enrichment and pathway analyses supported a broad cutaneous transcriptomic perturbation in CPUO. CPUO vs. HC showed 274 significant GO BP terms, 12 GO MF terms, nine GO CC terms, 12 KEGG pathways, and two Reactome pathways. AD vs. HC showed 11 GO BP terms, five GO MF terms, seven GO CC terms, four KEGG pathways, and five Reactome pathways. CPUO vs. AD did not yield significant over-representation terms at the primary DEG threshold, consistent with the small number of significant genes in that contrast. Because AD vs. HC produced more DEGs (1640) yet fewer enriched terms than CPUO vs. HC (1002 DEGs), we re-examined this apparent paradox and confirmed that it is not an artifact of the background gene set: an identical universe of 13,178 Entrez-mappable genes, derived from the same 20,595 tested genes, was supplied to every contrast. The difference is one of breadth rather than strength. AD enrichment was in fact substantially stronger at the top of the ranking but very narrow, being dominated by a tightly overlapping family of epidermal-differentiation terms (intermediate filament organization, adjusted *p* = 1.9 × 10^−10^; keratinization, adjusted *p* = 8.8 × 10^−9^). CPUO enrichment was weaker at its maximum (gland development and angiogenesis, adjusted *p* = 1.9 × 10^−5^) but distributed across a wider range of processes, including angiogenesis, epithelial and branching morphogenesis, intermediate filament organization, cornified envelope formation, positive regulation of cell migration, MAPK and PI3K-Akt signaling, and cytokine-cytokine receptor interaction (Supplementary Figure S2). The number of significant terms therefore indexes the diversity, not the magnitude, of enrichment, and should not be read as evidence that AD skin is less perturbed than CPUO skin. These results support epidermal-stress, vascular/remodeling, and immune-signaling involvement in CPUO, while the broader neuroimmune-stromal niche model remains a framework requiring integrated support from the analyses that follow (Figure 1).

### 3.2. An XOmiVAE-Inspired Latent Model Identifies Interpretable Transcriptional Programs Associated with CPUO

**Figure 2:**
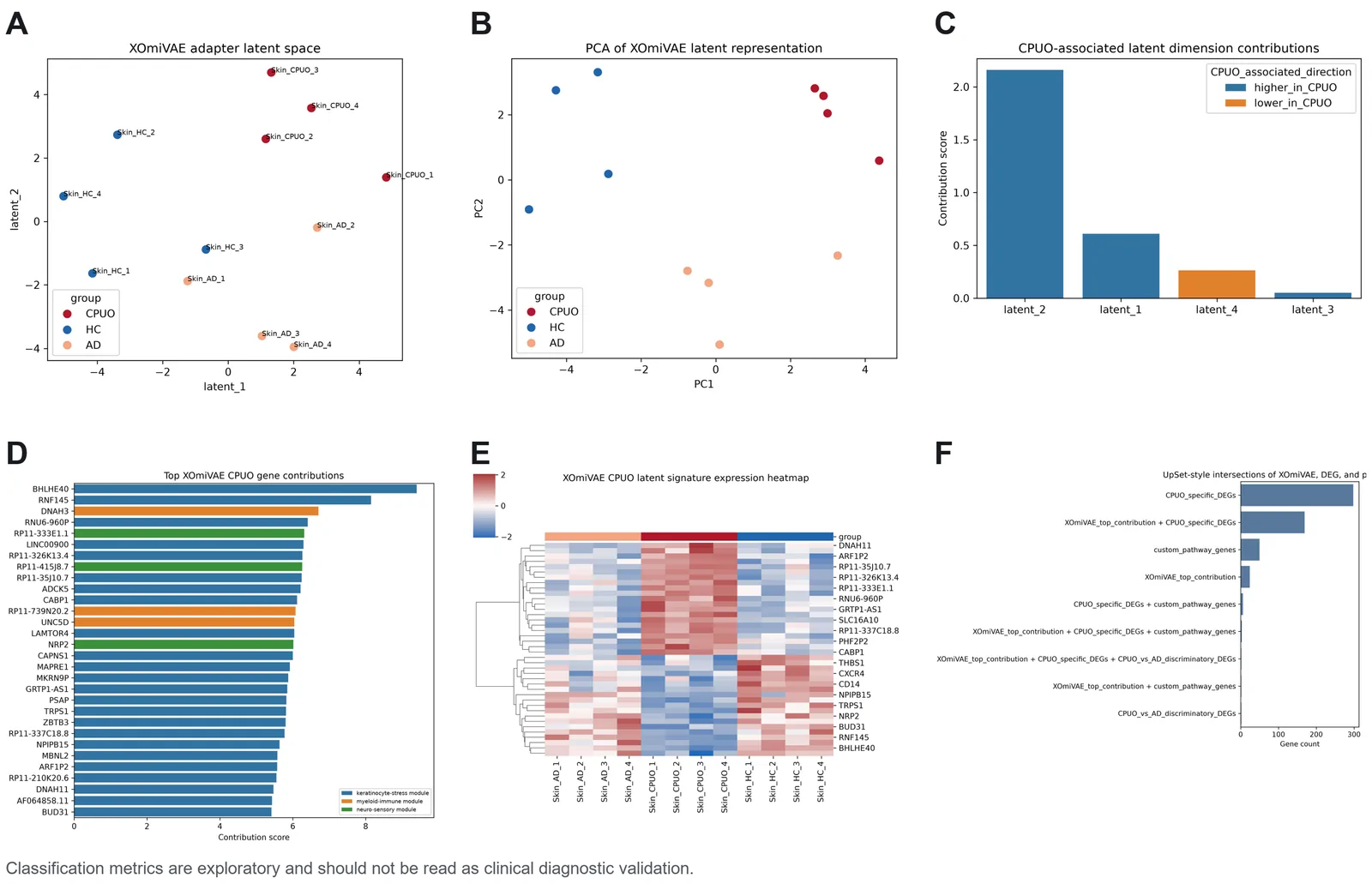
XOmiVAE-inspired interpretable latent transcriptomic state. An expression-only, mono-omics adaptation was applied to 2000 selected genes from the GSE237920 bulk expression matrix using a four-dimensional latent space. (**A**) Distribution of the 12 samples in latent dimensions 1 and 2, colored by diagnostic group. (**B**) Principal component analysis of the four-dimensional latent representation, colored by diagnostic group. (**C**) Contribution scores of the four latent dimensions to the CPUO-associated latent state; colors indicate whether each latent dimension was higher or lower in CPUO. Latent_2 showed the largest contribution, followed by latent_1 and latent_4. (**D**) The 30 genes with the largest model-derived gene-level contribution scores; bar colors indicate their assigned functional modules. (**E**) Row-wise z-standardized expression heatmap of the top 40 CPUO-driving genes across the 12 samples; columns are grouped by diagnosis, rows are hierarchically clustered, and blue and red indicate lower and higher standardized expression, respectively. (**F**) UpSet-style bar plot showing gene-count intersections among the top-decile XOmiVAE gene contributions, CPUO-versus-HC-specific differentially expressed genes, CPUO-versus-AD discriminatory genes, and the predefined pathway-gene set. Post hoc feature attribution nominated 201 CPUO-driving genes across the keratinocyte-stress, myeloid-immune, neu-ro-sensory, and stromal-vascular modules. The classification analyses are exploratory because the cohort contains only 12 samples and should not be interpreted as clinical diagnostic validation; the corresponding receiver operating characteristic curves are presented in Supplementary Figure S3.

An XOmiVAE-inspired, expression-only adaptation was run as the primary deep learning model in mono-omics mode using an adapter wrapper for the GSE237920 bulk matrix. The model used 2000 selected input genes and a VAE-style encoder/decoder with a supervised classification head, and was implemented using a PyTorch backend. The adaptation retains the architecture of the original XOmiVAE but replaces its native DeepSHAP explanation module with reproducible post hoc contribution metrics, and omits its multi-omics input branches; we therefore refer to it throughout as an XOmiVAE-inspired adaptation rather than as XOmiVAE itself, and enumerate the differences in Methods. The purpose of the analysis was to identify an interpretable CPUO-associated latent transcriptomic state, not to construct a clinically validated diagnostic model (Figure 2).

In exploratory leave-one-out evaluation under the protocol used in the original analysis, the model separated CPUO from HC with an accuracy, balanced accuracy, and ROC AUC of 1.00 across eight samples, and gave the same values for CPUO versus AD across eight samples. These values are reported here only as the comparator for the nested analysis in Section 3.9, because that protocol is not free of label information. Input-gene selection was repeated inside every fold using only the 11 (or 7) training samples, but the fold-internal selection prioritized a list of CPUO-versus-HC-enriched and CPUO-versus-AD differentially expressed genes derived from all 12 samples, so 480 of the 700 features chosen in each fold were selected with the held-out sample contributing to the choice. When the differential expression step is itself moved inside the fold, leave-one-out AUC remains 1.000 ± 0.000 for CPUO versus HC but falls to 0.741 ± 0.031 for CPUO versus AD (Section 3.9). With four samples per group, a leave-one-out accuracy of 1.00 indicates that the discovery cohort is completely separable and provides no estimate of generalization; ROC curves for these contrasts are therefore reported in Supplementary Figure S3 rather than as a main result. In the exploratory three-class task (HC vs. AD vs. CPUO), leave-one-out accuracy and balanced accuracy were both 0.833 across 12 samples, with CPUO and AD contributing the observed misclassifications while all HC samples were correctly classified. Repeated stratified cross-validation on the final latent representation yielded a mean accuracy and balanced accuracy of 1.00 across 100 repeats; because it reuses a latent representation fitted on all 12 samples, this is an internal stability check and not independent validation (Figure 2; Supplementary Figure S3).

The latent representation highlighted CPUO-associated transcriptional structure beyond standard dimensionality reduction. Among latent dimensions, latent_2 showed the strongest CPUO-associated contribution, with a mean latent value of 3.066 in CPUO versus −1.080 in non-CPUO samples, an effect size of 2.436, and a latent contribution score of 2.159. Latent_1 also showed higher values in CPUO, with a mean of 2.458 in CPUO versus −1.090 in non-CPUO samples, an effect size of 1.626, and a contribution score of 0.609. Latent_4 was lower in CPUO, with a mean of −1.069 in CPUO versus 0.561 in non-CPUO samples, an effect size of −0.852, and a contribution score of 0.263. These findings support a compact latent transcriptomic state associated with CPUO in this dataset (Figure 2).

### 3.3. Latent Feature Attribution Prioritizes Candidate CPUO-Driving Neuroimmune-Stromal Genes

Feature attribution was performed using reproducible post hoc contribution metrics, because direct tensor-level DeepSHAP derivations were not accessible for the adapted model architecture. Gene-level contribution scores combined linearized encoder-to-latent model weights, CPUO-associated latent/classifier contribution, observed gene-latent correlations, and CPUO expression effect size. These contribution scores were then integrated with CPUO-versus-HC-enriched DEG evidence, CPUO-vs-AD discriminatory DEG evidence, and pathway-gene evidence. Genes were assigned to one of four interpretable modules using a two-stage rule described in Methods, and the module assignment and the basis on which it was made are reported for every gene in Supplementary Table S2 (Figure 2).

The final CPUO-driving signature contained 201 genes distributed across four interpretable modules: 128 keratinocyte-stress genes, 58 myeloid-immune genes, 13 neuro-sensory genes, and two stromal-vascular genes. The top-ranked CPUO-driving genes included *BHLHE40*, *NRP2*, *CD14*, *TXNRD1*, *CXCL1*, *NRP1*, *CXCR4*, *NTRK2*, *RNF145*, *THBS1*, *DNAH3*, and *CABP1* (Table 1). Eleven genes had support from two independent evidence categories: *BHLHE40*, *NRP2*, *PHF2P2*, *CD14*, *TXNRD1*, *CXCL1*, *NRP1*, *CXCR4*, *NTRK2*, *THBS1*, and *MCAM*. Three caveats attach to this ranking and are important for its interpretation. First, 51 of the 201 entries correspond to pseudogenes, lncRNA clone identifiers or other non-coding features—for example *PHF2P2*, *RNU6-960P*, *LINC00900*, and multiple *RP11*-clone-based identifiers. *PHF2P2*, which ranks third by driving score, is an annotated pseudogene (the HGNC ‘P2’ suffix denotes pseudogene status), and its assignment to a neuro-sensory program has no biological basis; it has been removed from the representative genes in Table 1 and is retained only in the complete Supplementary signature table. Second, among protein-coding entries not previously linked to itch, two are biologically coherent with the proposed niche model: *THBS1* encodes thrombospondin-1, a matricellular activator of latent TGF-beta and a documented mediator of fibroblast-macrophage crosstalk, and *CXCR4* is expressed by both myeloid cells and sensory neurons and is the receptor for keratinocyte- and fibroblast-derived CXCL12, which we evaluate as a candidate skin-to-DRG axis below. Third, *DNAH3* (an axonemal dynein heavy chain), *CABP1*, *TRIM7*, and *FRAT2* also appear among the highest-scoring genes but have no established cutaneous or sensory function; all four were assigned to modules by expression correlation rather than by any curated annotation, and each is supported by a single evidence category, and we regard them as unexplained and most plausibly noise-driven at this cohort size. These results highlight a CPUO-associated latent signature spanning keratinocyte stress, immune/myeloid activity and neuro-sensory signaling, with only a token stromal-vascular component (Figure 2). The stability of this list is limited, and is quantified in Section 3.9. Across 1000 bootstrap resamples in which the whole derivation chain was repeated, the median Jaccard overlap with the 201 genes reported here was 0.11, and only six genes reached a selection frequency of at least 80%: *NRP2* (0.974), *BHLHE40* (0.957), *CAPNS1* (0.922), *DNAH11* (0.871), *TRPS1* (0.832), and *NPM3* (0.808). Five of the six belong to the 201-gene list reported here; *NPM3* does not appear in it at all. *NRP2* and *BHLHE40* are the two highest-scoring genes in Table 1, so the top of the ranking is reproducible while the tail is not, and interpretation at the level of individual genes should be confined to that stable core [29]. Because that core is what we ask readers to take seriously, its members warrant explicit comment. *NRP2* (neuropilin-2) is a co-receptor for class-3 semaphorins and for VEGF-C, expressed both on sensory axons and on lymphatic and vascular endothelium, so its module label is a naming convention rather than a functional claim (Section 2.5). *BHLHE40* is a hypoxia-, circadian- and immune-responsive transcriptional repressor active in keratinocytes and T cells, and is also one of the four genes separating CPUO from AD in the direct contrast. *TRPS1* is a *GATA*-type transcription factor required for hair follicle and epidermal appendage development, which is consistent with an epidermal-stress-dominated state. The remaining three are not interpretable in this context: *CAPNS1* encodes the ubiquitously expressed small regulatory subunit shared by calpain-1 and calpain-2; *NPM3* is a nucleoplasmin-family histone chaperone; and *DNAH11* is an axonemal dynein heavy chain associated with motile-cilium function, with no established role in skin or sensory biology. *DNAH11* is a different gene from the *DNAH3* discussed above, and the appearance of two unrelated axonemal dyneins among both the highest-scoring and the most frequently reselected genes is more consistent with a systematic artifact of this platform and cohort than with biology. We therefore treat the stable core as reproducible under resampling without treating all of its members as biologically interpretable.

An auxiliary SHAP sensitivity analysis was performed using logistic regression, random forest, and linear SVM classifiers, because tensor-level DeepSHAP attribution could not be computed for the adapted model itself and could only be applied to surrogate classifiers trained on the same input matrix. Across the top 10, 20, 50, and 100 SHAP-ranked genes, each auxiliary model showed zero overlap with the 201-gene signature. We report this as a negative result rather than as corroboration: independent model families applied to the same data do not recover the signature, and this is the clearest single constraint on the robustness of the gene-level nominations. The SHAP rankings were not used to redefine the primary signature; the reasons why we nevertheless consider the comparison only partially informative, and the consequences for how the signature should be read, are set out in the Section 4 (Supplementary Figure S4). Read together with the bootstrap results in Section 3.9, this places the gene-level nominations at the weakest end of the evidence presented in this study.

### 3.4. Peripheral Blood scRNA-Seq Evaluates the Systemic Detectability of the CPUO Skin Latent Signature

Public GSE295457 peripheral blood mononuclear cell (PBMC) scRNA-seq data were processed to evaluate whether the CPUO skin latent signature was detectable within circulating immune compartments. The processed PBMC dataset comprised 75,161 cells from eight public GSE295457 samples, categorized at the GEO sample level into four CPUO and four healthy control (HC) samples; following quality control, 36,131 CPUO cells and 39,030 HC cells were retained. No PBMC samples from patients with atopic dermatitis were available in this dataset, so the peripheral analysis is restricted to the CPUO-versus-HC contrast.

**Figure 3:**
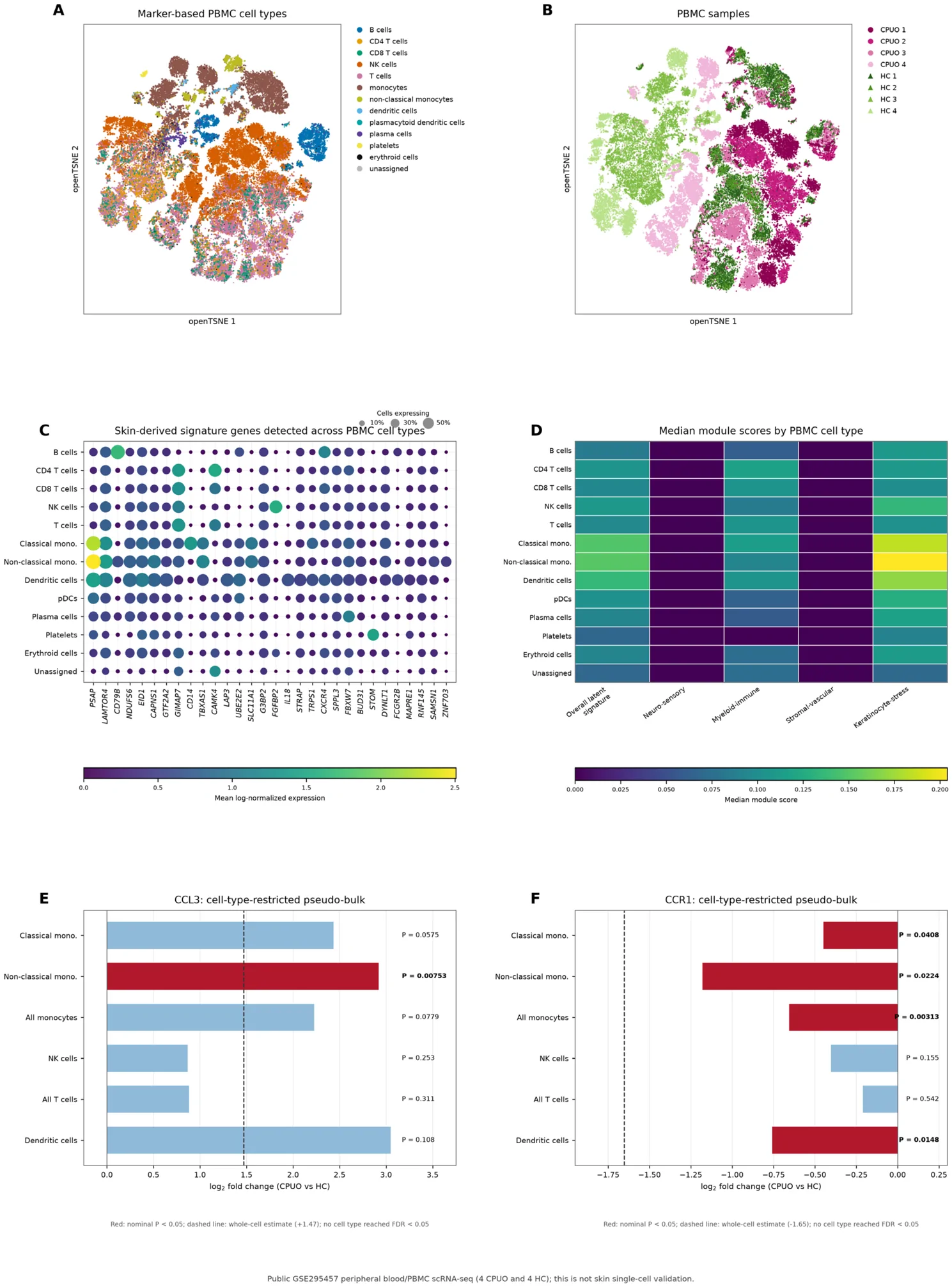
CPUO peripheral blood single-cell RNA-sequencing analysis. Public GSE295457 peripheral blood/PBMC single-cell RNA-sequencing data from four patients with chronic pruritus of unknown origin (CPUO) and four healthy controls (HC) were processed, yielding 75,161 quality-control-retained cells. (**A**) Two-dimensional openTSNE embedding colored by marker-based PBMC cell-type annotation. Each of the 13 displayed cell-type cat-egories is represented by a distinct color. (**B**) The same openTSNE embedding colored by sample; circles denote CPUO samples and triangles denote HC samples. (**C**) Expression of the 30 most widely detected genes from the 201-gene skin-derived CPUO latent signature across the annotated PBMC cell types. Genes were ranked by their maximum fraction of expressing cells across cell types; dot size indicates the fraction of cells expressing the gene, and color indicates mean log-normalized expression. (**D**) Heatmap of the median per-cell module score in each PBMC cell type for the overall XOmiVAE-derived CPUO latent signature and the neuro-sensory, myeloid-immune, stromal-vascular, and keratinocyte-stress modules. Module scores were calculated as the mean log-normalized ex-pression of the detected genes in each module. (**E**) Cell-type-restricted, sample-level pseudo-bulk log2 fold-change estimates for CCL3 in CPUO versus HC. (**F**) Corresponding estimates for CCR1. In panels E and F, red bars indicate nominal *p* < 0.05, blue bars indicate nominal *p* ≥ 0.05, annotated values are nominal *p*-values, and the vertical dashed line indicates the whole-cell pseudo-bulk estimate. No gene reached an FDR-adjusted *p*-value < 0.05 within any displayed cell type; these cell-type-restricted results are exploratory. The GSE295457 data represent peripheral blood/PBMC profiling and do not constitute skin single-cell validation.

Marker-based cell-type annotation identified natural killer (NK) cells (25,849 cells), T cells (22,521), monocytes (13,226), B cells (4665), CD4+ T cells (2472), CD8+ T cells (1824), non-classical monocytes (1495), unassigned cells (1378), plasma cells (873), dendritic cells (304), platelets (289), plasmacytoid dendritic cells (154), and erythroid cells (111). Quality-control metrics, clustering, and the marker genes underlying these assignments are shown in Supplementary Figure S5 (Figure 3; Supplementary Figure S5).

Among the 201 CPUO signature genes, 154 were detected in the PBMC dataset. Detectability at the module level varied across transcriptional programs: 10 of 13 neuro-sensory, 52 of 58 myeloid-immune, 2 of 2 stromal-vascular, and 90 of 128 keratinocyte-stress module genes were identified in PBMCs. The 47 undetected entries were dominated by non-coding features: 33 were lncRNA clone identifiers or pseudogene-like symbols, and the remainder were transcripts with tissue-restricted expression that would not be expected in circulating leukocytes, such as *LOR* (loricrin), *ACKR1*, and *CCL21*. Detectability in this sense states which skin-derived transcripts are measurable in blood at all; it says nothing about whether they differ between groups, which is addressed next (Figure 3; Supplementary Figure S6).

Given that GSE295457 contains both CPUO and HC PBMC samples, we additionally performed a group-aware pseudo-bulk comparison (CPUO vs HC), using GEO samples rather than individual cells as the statistical units. Out of 18,318 genes tested, only one PBMC gene met the threshold for significance (FDR < 0.05 and |log2 fold change| > 0.5): *FKBP15* was significantly downregulated in CPUO compared to HC (log2 fold change = −0.872, FDR = 0.0115). Using a nominal *p* < 0.05 and |log2 fold change| > 0.5, 935 differentially expressed genes were observed, comprising 303 upregulated and 632 downregulated genes in CPUO; these nominal results were considered exploratory given *n* = 4 per group. No PBMC gene overlapping with the skin-derived signature retained significance following FDR correction, although nominal exploratory overlaps included *NRP2*, *RNASE1*, *SLC25A32*, *CXCR4*, *TRIM7*, *BHLHE40*, *FRAT2*, *CD14*, *GIMAP7*, and *SH2D2A*. Sample-level cell-type composition analysis revealed no FDR-significant differences across immune compartments. This whole-cell analysis therefore indicates that the skin-derived signature has limited detectability as an aggregate peripheral signal. As shown next, that limitation is a property of whole-cell aggregation and should not be generalized to peripheral CPUO biology (Figure 3).

Because whole-cell pseudo-bulk aggregation averages across all circulating populations and can mask signals confined to a single compartment, we repeated the CPUO-versus-HC comparison within individual cell types, reconstructing sample-by-cell-type pseudo-bulk profiles directly from the raw GSE295457 count matrices and restricting them to the same QC-retained cells. We focused on monocyte and NK compartments because the primary report of this dataset identified *CCL3* upregulation in these populations and linked CCL3 to sensory neuron sensitization through CCR1 [25]. *CCL3* was higher in CPUO in every myeloid compartment examined: log2 fold change +2.43 in classical monocytes (*p* = 0.057; 3653 CPUO and 9573 HC cells), +2.92 in non-classical monocytes (*p* = 0.0075; 539 and 956 cells), +3.05 in dendritic cells (*p* = 0.108), and +2.23 in monocytes overall (*p* = 0.078). NK cells showed the same direction with a smaller effect (+0.87, *p* = 0.25; 15,523 CPUO and 10,326 HC cells). By comparison, the whole-cell pseudo-bulk estimate for *CCL3* was +1.47 (*p* = 0.094, FDR = 0.70), approximately half the monocyte-restricted effect. *CCR1*, the receptor through which CCL3 has been proposed to sensitize sensory neurons, was consistently lower in CPUO myeloid cells (−0.45 in classical monocytes, *p* = 0.041; −1.18 in non-classical monocytes, *p* = 0.022; −0.66 in monocytes overall, *p* = 0.0031), a pattern compatible with receptor downregulation under sustained ligand exposure but not testable in this dataset. With four samples per group, no gene reached FDR significance in any individual cell type, so all cell-type-restricted results are nominal and exploratory. Nonetheless, the direction and the relative magnitude of the *CCL3* effect reproduce the published finding and indicate that our whole-cell analysis had diluted, rather than contradicted, a compartment-restricted signal (Figure 3; Supplementary Table S3).

### 3.5. Atlas-Guided Projection onto a Public Skin Spatial Reference Locates Signature Modules Within Skin Architecture

**Figure 4:**
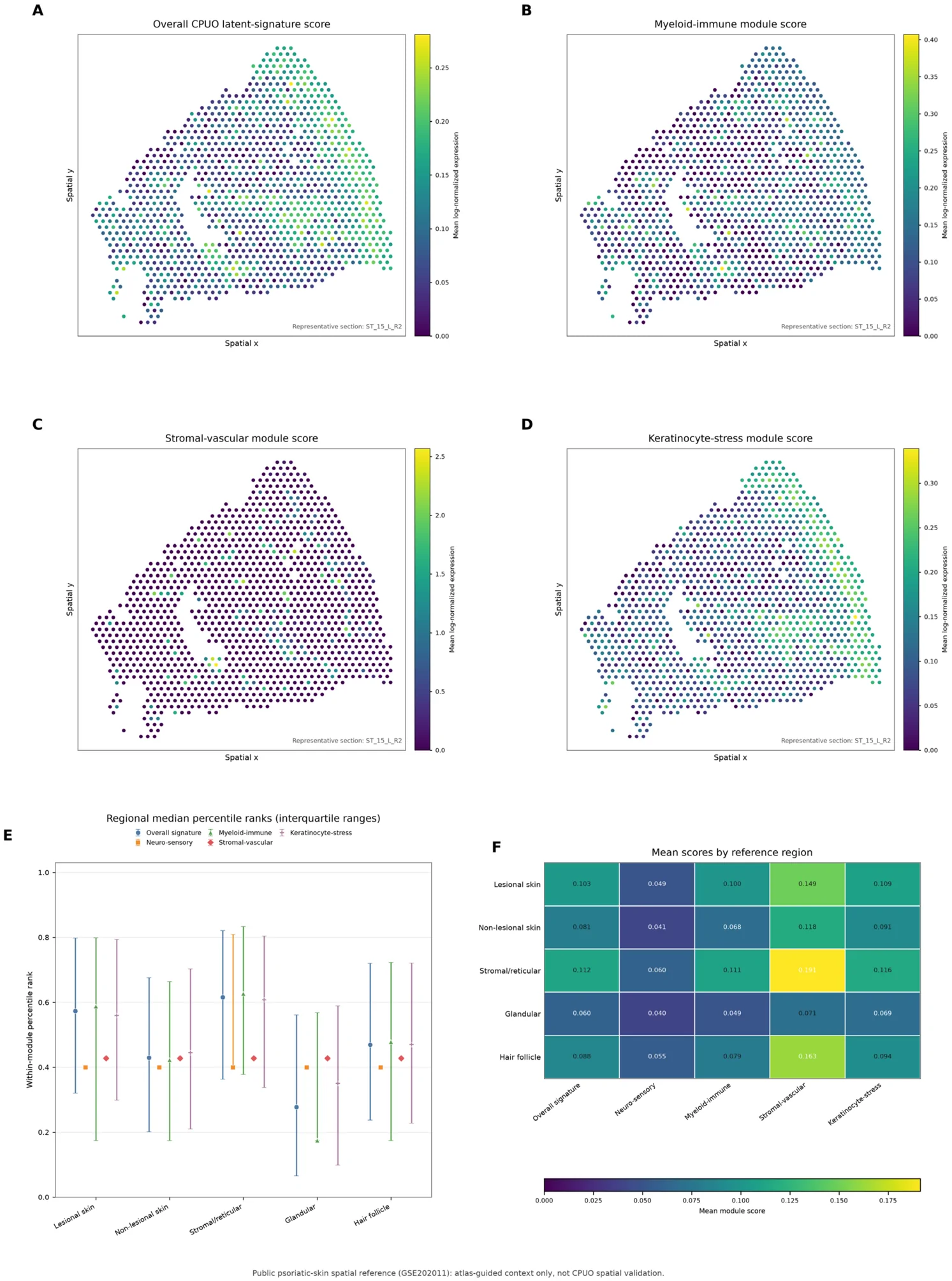
Atlas-guided spatial neighborhood inference. The public GSE202011 psoriatic-skin spatial transcriptomic reference was used to provide atlas-guided context and does not constitute spatial validation in patients with chronic pruritus of unknown origin (CPUO). The processed reference comprised 24,227 spots across five available region labels; “lesional” and “non-lesional” refer to psoriatic skin. (**A**) Spatial distribution of the overall XOmiVAE-derived CPUO latent-signature score in the representative lesional reference section ST_15_L_R2, comprising 1183 spots. (**B**) Spatial distribution of the myeloid-immune module score in the same representative section. (**C**) Spatial distribution of the stromal-vascular module score in the same representative section. (**D**) Spatial distribution of the keratinocyte-stress module score in the same representative section. In panels (**A**–**D**), color represents the mean log1p-transformed expression of the signature genes detected in the reference dataset after normalization to 10,000 counts per spot. (**E**) Regional summaries of the overall signature and four component-module scores across all 24,227 spots. Each score was converted to a percentile rank across all spots before regional summarization; symbols represent medians, and vertical bars represent interquartile ranges. (**F**) Heatmap showing the untransformed regional mean scores, with the corresponding values annotated in each cell. Overall signature coverage was 154 of 201 genes; module-level coverage was 8 of 13 neuro-sensory genes, 54 of 58 myeloid-immune genes, 2 of 2 stromal-vascular genes, and 90 of 128 keratinocyte-stress genes. Because the reference was derived from psoriatic skin, these patterns should be interpreted as reference-guided localization and generic inflammatory or epidermal-stress context rather than evidence of a CPUO-specific spatial niche.

A public skin spatial transcriptomic reference (GSE202011) was used for atlas-guided spatial inference of the CPUO signature. This reference is a psoriatic-disease spatial transcriptomics resource comprising matched lesional and non-lesional psoriatic samples together with healthy control samples [17]; the terms ‘lesional skin reference’ and ‘non-lesional skin reference’ below therefore denote psoriatic lesional and non-lesional skin, not CPUO tissue. The analysis processed 24,227 spatial spots/cells using available spatial neighborhood annotations and should be considered an inferred projection onto a different inflammatory dermatosis rather than direct spatial transcriptomic validation in CPUO patients. The annotated regions included lesional skin reference (9023 spots/cells), non-lesional skin reference (6305), hair follicle (4944), stromal/reticular dermis (2099), and glandular regions (1856) (Figure 4).

Signature coverage in the public spatial reference was robust, detecting 154 of the 201 (76.6%) genes comprising the overall CPUO niche score. Module-level coverage rates were similarly robust: 8 of 13 neuro-sensory genes (61.5%), 54 of 58 myeloid-immune genes (93.1%), 2 of 2 stromal-vascular genes (100%), and 90 of 128 keratinocyte-stress genes (70.3%). Mean overall signature scores varied across the evaluated anatomical regions, peaking at 0.103 in the lesional skin reference, followed by 0.088 in the hair follicle, 0.081 in the non-lesional skin reference, and 0.060 in the glandular region. Because the highest-scoring region is psoriatic lesional skin, this gradient is most conservatively read as showing that the signature tracks generic inflammatory and epidermal-stress activity in skin rather than a CPUO-specific spatial niche. Perivascular and stromal-immune remodeling in CPUO therefore remains an inferred interpretation pending direct CPUO spatial validation (Figure 4; Supplementary Figure S7).

### 3.6. Fibroblast and Myeloid Marker Programs Are Correlated but Do Not Differ Significantly Between Groups

**Figure 5:**
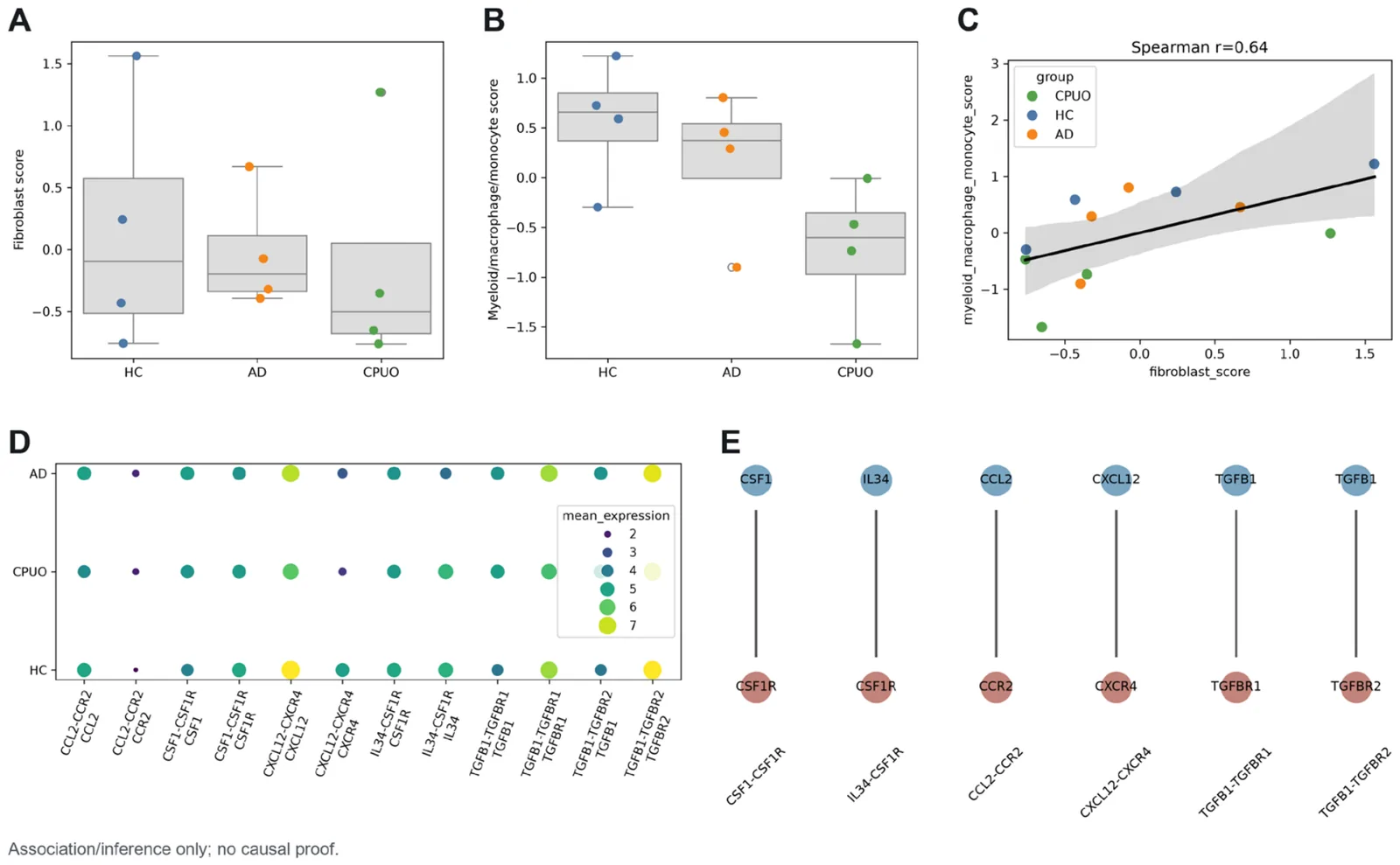
Fibroblast and myeloid marker programs in CPUO skin. Fibroblast and my-eloid/macrophage/monocyte marker scores were calculated in the GSE237920 bulk skin transcriptomic cohort by z-standardizing the expression of each detected marker gene across the 12 samples and then averaging the standardized values within each marker set. The cohort comprised healthy controls (HC; *n* = 4), patients with atopic dermatitis (AD; *n* = 4), and patients with chronic pruritus of unknown origin (CPUO; *n* = 4). (**A**) Fibroblast marker scores across HC, AD, and CPUO samples. (**B**) Myeloid/macrophage/monocyte marker scores across the three groups. In panels (**A**,**B**), boxes show the median and interquartile range, whiskers extend to the most extreme observations within 1.5 times the interquartile range, and colored points represent individual samples (HC, blue; AD, orange; CPUO, green). Group differences were evaluated using two-sided Kruskal–Wallis tests and were not significant for either the fibroblast score (*p* = 0.668) or the myeloid/macrophage/monocyte score (*p* = 0.092). (**C**) Association between the fibroblast and myeloid/macrophage/monocyte scores across all 12 samples. The black line shows the ordinary least-squares fit and the grey band shows its 95% confidence interval; point colors denote the diagnostic groups. The scores were positively correlated (Pearson r = 0.590, *p* = 0.043; Spearman r = 0.636, *p* = 0.026). (**D**) Mean normalized bulk-skin expression of the genes comprising six candidate ligand–receptor axes—*CSF1*–*CSF1R*, *IL34*–*CSF1R*, *CCL2*–*CCR2*, CXCL12–*CXCR4*, *TGFB1*–*TGFBR1*, and *TGFB1*–*TGFBR2*—in each diagnostic group. Dot size and color both indicate mean expression across the four samples in each group. (**E**) Schematic representation of the same candidate ligand–receptor pairs; blue nodes denote ligands, salmon nodes denote receptors, and connecting lines denote the queried pairs. Panel (**E**) does not encode interaction strength, directionality, or experimentally demonstrated cell–cell communication. All 16 fibroblast markers and 13 of 16 myeloid markers were detected in the bulk expression matrix. These analyses show bulk-tissue associations and candidate communication axes and do not establish causal fibroblast–myeloid regulation.

Fibroblast and myeloid/macrophage/monocyte programs were inferred from the bulk skin transcriptomic dataset using curated marker scores. All 16 fibroblast marker genes and 13 of the 16 myeloid lineage marker genes were detected in the expression profile; two myeloid markers also belong to the 201-gene CPUO signature, so the myeloid marker program and the myeloid-immune signature module are not fully independent. Mean fibroblast scores across the HC, AD, and CPUO groups were 0.154, −0.030, and −0.124, respectively, and mean myeloid/macrophage/monocyte scores were 0.560 in HC, 0.161 in AD, and −0.721 in CPUO. From these, we defined a single fibroblast-myeloid dominance score as the z-scored fibroblast score minus the z-scored myeloid score, so that positive values indicate relative fibroblast predominance; mean values were −0.491 (HC), −0.241 (AD), and +0.731 (CPUO). The marker scores used in this paragraph and in Figure 5 are z-transformed mean expression values. The version of the dominance score that enters the composite niche score is computed from percentile-rank marker scores instead, for the transportability reason given in Section 2.9; on that scaling, the group means are −0.320 (HC), −0.087 (AD) and +0.408 (CPUO), and the component-level statistics reported in Section 3.8 are those of the rank version. The two scalings agree in direction and differ in magnitude, and neither reaches significance (Figure 5).

Statistical evaluation did not reveal a significant difference in fibroblast scores across the three groups (Kruskal–Wallis *p* = 0.668). Myeloid lineage scores showed a non-significant trend (Kruskal–Wallis *p* = 0.092), with the CPUO versus HC comparison approaching marginal significance (Mann–Whitney *p* = 0.057). The fibroblast-myeloid dominance score did not differ significantly across groups (Kruskal–Wallis *p* = 0.167; CPUO versus HC *p* = 0.114), although the effect size was moderate and consistent in direction (Cliff’s delta +0.75 versus HC and +0.62 versus AD); on the percentile-rank scaling used inside the composite score the same comparison gives Kruskal–Wallis *p* = 0.500 and Cliff’s delta +0.50 versus HC and +0.25 versus AD. Across all 12 samples, fibroblast and myeloid scores were positively correlated (Pearson r = 0.590, *p* = 0.043; Spearman r = 0.636, *p* = 0.026). We therefore report a coordinated fibroblast-myeloid relationship at the bulk transcriptomic level together with a directionally consistent but statistically non-significant group shift; no causal or regulatory claim is supported by these data (Figure 5).

Key candidate fibroblast-myeloid ligand-receptor axes—including *CSF1-CSF1R*, *IL34-CSF1R*, *CCL2-CCR2*, CXCL12*-CXCR4*, and *TGFB1-TGFBR1*/*TGFBR2*—were further investigated. The public skin spatial reference (GSE202011) was used strictly to provide atlas-guided anatomical context for the detectability of these ligand-receptor pairs, and because that reference is a psoriatic-disease dataset, it establishes where such pairs are co-detectable in inflamed skin generally rather than in CPUO. Together, the marker-scoring and ligand-receptor analyses support the inferred stromal-myeloid component of the CPUO niche model, with the explicit caveat that the current bulk transcriptomic evidence remains correlational rather than causal (Figure 5; Supplementary Figure S8).

### 3.7. Projection onto a Public Human DRG Atlas Provides Cellular Context for Candidate Skin-to-Neuron Axes

**Figure 6:**
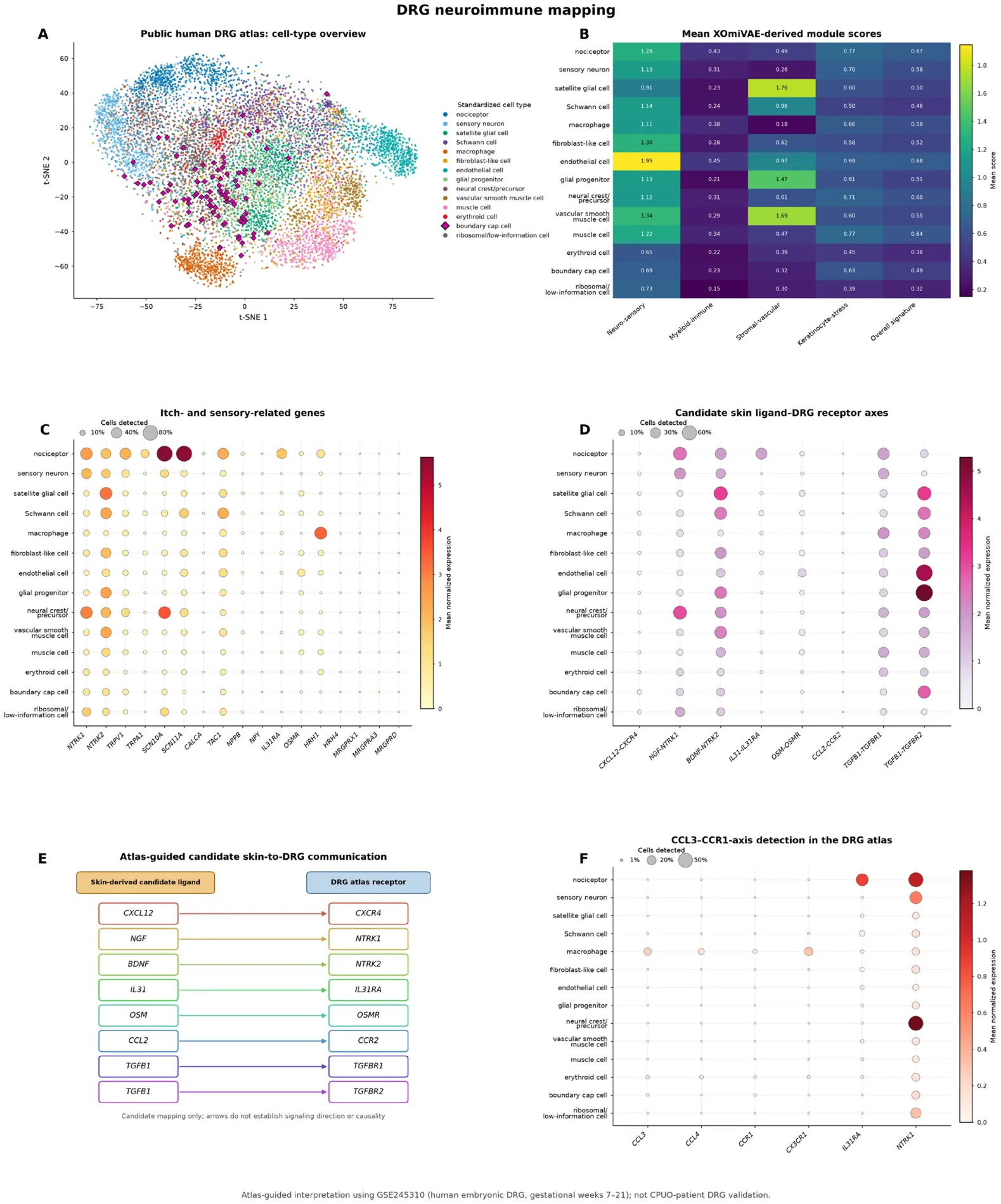
DRG neuroimmune mapping. (**A**) Stratified t-SNE overview of 11,461 cells sampled from the processed public human dorsal root ganglion (DRG) atlas (GSE245310), colored by 14 standardized cell-type labels. Boundary cap cells are shown as high-contrast magenta diamonds with black outlines and were plotted last to ensure that this rare population remains visible. (**B**) Heatmap of the mean XOmiVAE-derived neuro-sensory, myeloid-immune, stromal-vascular, keratinocyte-stress, and overall CPUO signature scores across the 14 DRG atlas cell types; values within cells are the unscaled mean scores. (**C**) Dot plot showing the expression of itch- and sensory-related genes across DRG atlas cell types. (**D**) Dot plot showing receptor expression for eight candidate skin ligand–DRG receptor axes across the same cell types. In panels C and D, dot size represents the percentage of cells in which the gene was detected, and color represents mean normalized expression. (**E**) Schematic summary of the eight atlas-guided can-didate skin-to-DRG ligand–receptor pairs. Arrows denote candidate mapping and do not establish signaling direc-tion or causality. (**F**) Focused dot plot of *CCL3*, *CCL4*, *CCR1*, *CX3CR1*, *IL31RA*, and *NTRK1* detection across standardized DRG cell types; dot size represents the percentage of cells in which the gene was detected, and color represents mean normalized expression. The reference atlas comprises 123,370 cells from human embryonic DRG at gestational weeks 7–21. These analyses provide atlas-guided neuroimmune context and do not constitute validation in DRG tissue from patients with CPUO.

CPUO signature modules were mapped onto a public human DRG/sensory neuron atlas (GSE245310) for atlas-guided neuroimmune interpretation. This atlas is a single-nucleus transcriptomic resource of human embryonic DRG sampled between gestational weeks 7 and 21 [26]; it is, to our knowledge, the most comprehensive publicly available human DRG single-cell resource, but it is developmental rather than adult tissue, and this constrains what can be inferred from it. The processed atlas contained 123,370 cells/spots across seven standardized cell-type labels used in the main figures: unannotated cells (48,001), sensory neurons (32,684), fibroblast-like cells (14,314), nociceptors (11,896), satellite glial cells (8895), Schwann cells (6949), and macrophages (631). This represents a reference-based projection onto developing human DRG rather than direct evidence of DRG transcriptional alterations in CPUO patients (Figure 6).

The public DRG atlas contained 148 of the 201 overall CPUO signature genes, corresponding to a coverage rate of 73.6%. Module-level coverage was comparable across modules: 8 of 13 neuro-sensory genes (61.5%), 51 of 58 myeloid-immune genes (87.9%), 2 of 2 stromal-vascular genes (100%), and 87 of 128 keratinocyte-stress genes (68.0%). The mean overall signature score was highest in nociceptors (0.748), followed by macrophages (0.614), fibroblast-like cells (0.596), and Schwann cells (0.536) among the annotated cell types. These expression patterns provide atlas-guided support for the involvement of sensory and neuroimmune compartments when interpreting CPUO-associated skin latent signatures, subject to the developmental-stage caveat noted above (Figure 6; Supplementary Figure S9).

Itch- and sensory-neuron-related genes showed the expected enrichment patterns within the DRG atlas populations. *SCN10A* and *SCN11A* were detected in 88.2% and 88.6% of nociceptors, respectively, with mean expression values of 6.122 and 6.074. *NTRK1* was detected in 50.9% of nociceptors, and *IL31RA* was present in 36.4%. *NTRK2* was detected in 53.8% of satellite glial cells, 43.8% of Schwann cells, and 36.5% of nociceptors, while *HRH1* was observed in 54.0% of macrophages and 23.3% of nociceptors. Candidate skin-to-DRG axes—including *CCL3-CCR1*, CXCL12*-CXCR4*, *NGF-NTRK1*, *BDNF-NTRK2*, *IL31-IL31RA*, *OSM-OSMR*, *CCL2-CCR2*, and *TGFB1-TGFBR1*/*TGFBR2*—were evaluated as putative atlas-guided interactions rather than validated mechanisms in CPUO patients. The *CCL3-CCR1* axis, which is the axis most directly implicated in CPUO by the primary report of the peripheral blood dataset we analyzed [25], was added to this list and queried explicitly. In this atlas, *CCR1* was largely confined to the resident myeloid compartment, being detected in 3.5% of DRG macrophages and in fewer than 0.5% of nociceptors, sensory neurons, satellite glial cells, and Schwann cells; *CCL3* itself showed the same distribution (13.5% of macrophages, <0.5% elsewhere). We do not interpret this as evidence against neuronal CCR1 signaling in CPUO. Chemokine receptors are expressed at low copy number and are poorly captured by single-nucleus sequencing, and this atlas profiles embryonic rather than adult ganglia, in which nociceptor receptor repertoires are still being specified. The observation does mean, however, that our data provide atlas-level support for a myeloid rather than a directly neuronal locus of *CCR1* expression, and that the neuronal arm of the *CCL3-CCR1* axis requires adult human DRG or functional data to test (Figure 6).

### 3.8. The CPUO Neuroimmune-Stromal Niche Score Separates CPUO from AD and Healthy Skin Within the Discovery Cohort

**Figure 7:**
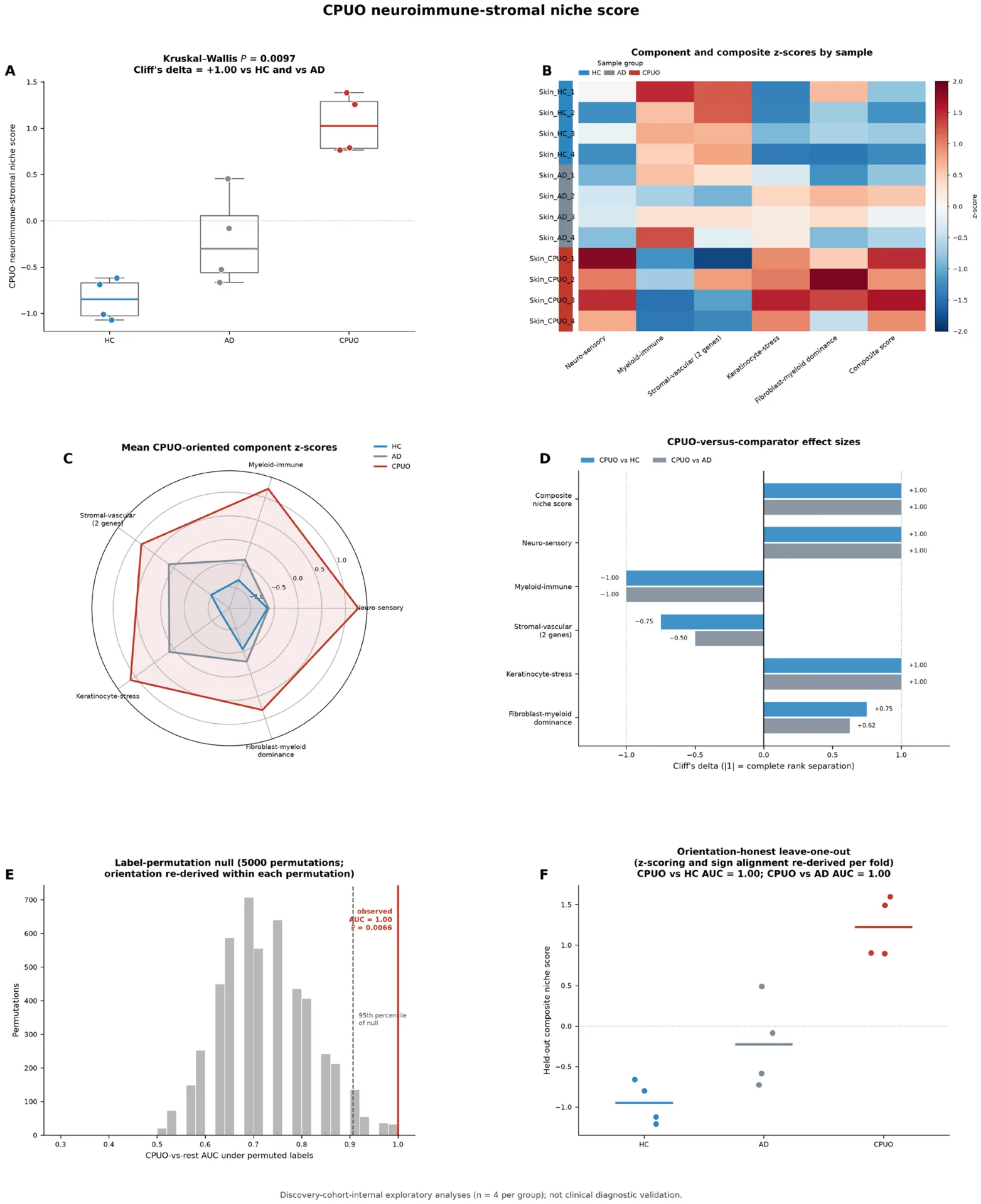
CPUO neuroimmune-stromal niche score. The composite score was calculated as the mean of five z-standardized, sign-aligned components: the XOmiVAE-derived neuro-sensory, myeloid-immune, stromal-vascular, and keratinocyte-stress module scores and the marker-based fibroblast-myeloid dominance score. The discovery cohort comprised healthy controls (HC; *n* = 4), patients with atopic dermatitis (AD; *n* = 4), and patients with chronic pruritus of unknown origin (CPUO; *n* = 4). (**A**) Distribution of the composite niche score across HC, AD, and CPUO samples. Boxes show the median and interquartile range, whiskers extend to the most extreme observations within 1.5 times the interquartile range, and points represent individual samples. The grey dotted horizontal line marks a niche score of zero. The three-group Kruskal–Wallis test gave *p* = 0.0097; Cliff’s delta was +1.00 for CPUO versus HC and +1.00 for CPUO versus AD. (**B**) Heatmap of the five component scores and the composite niche score across individual samples. Each column was z-standardized across the 12 samples; the colored strip beside the rows denotes sample group. (**C**) Radar plot of the mean CPUO-oriented component z-scores in each group. Component signs were aligned so that positive values indicate the CPUO-associated direction within the discovery cohort. (**D**) Cliff’s delta effect sizes for the composite score and each component in CPUO versus HC and CPUO versus AD. Positive values indicate higher values in CPUO, negative values indicate lower values in CPUO, and |delta| = 1 denotes complete rank separation. (**E**) Null distribution of CPUO-versus-rest AUC values obtained from 5000 label permutations, with component orientation re-derived within each permutation. The red line marks the observed AUC of 1.00, and the grey dashed line marks the 95th percentile of the permutation null distribution; the permutation *p* value was 0.0066. (**F**) Held-out composite scores from orientation-honest leave-one-out analysis, in which z-scoring and component sign alignment were re-derived within each training fold. Points represent held-out samples and horizontal colored lines denote group means. The grey dotted horizontal line marks a niche score of zero. The held-out AUC was 1.00 for CPUO versus HC and 1.00 for CPUO versus AD. All classification analyses are exploratory and discovery-cohort-internal and should not be interpreted as clinical diagnostic validation.

The CPUO neuroimmune-stromal niche score was constructed from the interpretable latent signature modules, incorporating neuro-sensory, myeloid-immune, stromal-vascular, and keratinocyte-stress programs alongside the fibroblast-myeloid dominance score defined above. All module genes used for the niche score were detected in the bulk expression dataset, achieving 100% coverage across all modules (13 neuro-sensory, 58 myeloid-immune, two stromal-vascular, and 128 keratinocyte-stress genes). Each of the five components was z-scored across the 12 samples and then sign-aligned so that higher composite values reflected the CPUO-associated direction; the consequences of performing this alignment in the same samples in which the score is evaluated are quantified below. Only the myeloid-immune and stromal-vascular components required sign inversion, both being lower in CPUO. The stromal-vascular component contains only two genes (*THBS1* and *MCAM*) and is not a module in any meaningful sense; it is reported throughout as exploratory and is not treated as comparable to the 128-gene keratinocyte-stress program (Figure 7).

The composite niche score was elevated in CPUO relative to both AD and HC. Mean scores were 0.996 in CPUO, −0.174 in AD and −0.822 in HC, with medians of 0.951, −0.314, and −0.862, respectively. CPUO was completely rank-separated from each comparator group: Cliff’s delta was +1.00 for CPUO versus HC and +1.00 for CPUO versus AD, with exact Mann–Whitney *p* = 0.0286 in both cases. That *p*-value is the smallest attainable in a two-sided 4-versus-4 test, so it records complete separation rather than effect magnitude; the accompanying Cliff’s delta is the informative quantity. AD and HC were not completely separated (Cliff’s delta +0.875, *p* = 0.0571). The three-group Kruskal–Wallis test gave an asymptotic *p* of 0.0097 (H = 9.27), which is above the 0.0073 floor of a 4/4/4 design because AD and HC overlap; the exact *p* obtained by enumerating all 34,650 label assignments is 5.2 × 10^−4^. Module-level comparisons gave asymptotic Kruskal–Wallis *p*-values of 0.0249 (neuro-sensory), 0.0183 (myeloid-immune), 0.0498 (stromal-vascular), 0.0073 (keratinocyte-stress), and 0.500 (fibroblast-myeloid dominance), with Cliff’s delta versus HC of +1.00, −1.00, −0.75, +1.00, and +0.50, respectively. After Benjamini–Hochberg correction across the five components, the keratinocyte-stress (adjusted *p* = 0.036), neuro-sensory and myeloid-immune (both 0.042) components remain significant, whereas the stromal-vascular (0.062) and fibroblast-myeloid (0.500) components do not (Figure 7). Exhaustive stratified bootstrap intervals for these effect sizes are as follows. For the components on which CPUO is completely rank-separated from a comparator—neuro-sensory, myeloid-immune, and keratinocyte-stress against both HC and AD, and the composite score itself—every one of the 65,536 possible resamples also shows complete separation, so the 95% interval is degenerate at the boundary (+1.00 to +1.00, or −1.00 to −1.00). This is a structural consequence of complete separation at four samples per group and is not a statement of precision. For the two components on which separation is incomplete the intervals are informative and wide: stromal-vascular delta = −0.75 (95% CI −1.00 to 0.00) versus HC and −0.50 (−1.00 to 0.50) versus AD, and fibroblast-myeloid dominance delta = +0.50 (−0.38 to 1.00) versus HC and +0.25 (−0.62 to 1.00) versus AD; both therefore include the null. Two pre-specified sensitivity analyses bear on how this composite should be read. Removing the fibroblast-myeloid dominance component leaves every CPUO contrast unchanged (Cliff’s delta +1.00 versus both HC and AD, *p* = 0.0286) but raises the AD-versus-HC separation to complete (delta +1.00, *p* = 0.0286), so the four-component score orders the three groups monotonically as HC < AD < CPUO; this is consistent with the reading developed in Section 3.10, in which the axis the score captures is inflammatory tone rather than a CPUO-specific state. Reassigning *NRP1* and *NRP2* from the neuro-sensory to the stromal-vascular module (Section 2.5) leaves the eleven-gene neuro-sensory component completely separating CPUO from both HC and AD (delta +1.00, asymptotic Kruskal–Wallis *p* = 0.024) and leaves the composite contrasts unchanged; the four-gene stromal-vascular component becomes stronger rather than weaker (delta −1.00 versus HC). The neuroimmune reading of the signature therefore does not depend on where the two neuropilins are filed. The uncertainty that matters for the composite score is not the group contrast given a fixed score, but the construction of the score itself, and that is quantified in Section 3.9.

Because the direction of each score component was aligned using the same 12 samples in which the score was then evaluated, conventional ROC statistics are circular in this setting; ROC curves are therefore presented in Supplementary Figure S10 rather than as a main result, and the AUC of 1.00 obtained for both CPUO versus HC and CPUO versus AD is reported there for completeness only. Two additional analyses bound how much of the observed separation is attributable to that circularity. First, we repeated leave-one-out evaluation with both z-scoring and component orientation re-derived within each training fold, so that the held-out sample never contributed to the orientation decision; held-out composite scores still separated the groups completely (AUC 1.00 for both contrasts), indicating that the orientation step is stable across resampling rather than driven by individual samples. Second, we performed a label-permutation test in which group labels were shuffled 5000 times, and the entire orientation-and-scoring procedure was repeated within each permutation; the observed separation was matched or exceeded in 0.66% of permutations (*p* = 0.0066), against a null mean AUC of 0.725 and a 95th percentile of 0.906. The niche score therefore captures structure that is unlikely to arise from random labeling of this cohort, but its apparent perfect accuracy remains a discovery-cohort-internal property that provides no estimate of external performance, and it should not be regarded as a validated clinical diagnostic tool (Figure 7; Supplementary Figure S10). Two qualifications must be attached to those two analyses. The fold-internal re-orientation left the upstream signature, the module definitions and the differential expression list unchanged, and the 5000-fold permutation likewise re-ran only the orientation-and-scoring procedure rather than the feature-discovery pipeline that produced the modules; neither therefore addresses the dominant source of optimism. Section 3.9 reports the analysis in which the entire pipeline, differential expression onward, is re-executed inside each fold and inside each permutation. Under that protocol, the composite score retains an AUC of 0.919 ± 0.079 against healthy skin but falls to 0.731 ± 0.068 against AD, and—as reported in Section 3.9—neither of those values exceeds its own fully nested permutation null. The 5000-fold permutation reported in this section should therefore not be read as establishing the composite score; it establishes only that the orientation-and-scoring step is not by itself sufficient to manufacture the observed separation.

### 3.9. Nested Re-Validation Confirms Separation from Healthy Skin but Not from Atopic Dermatitis

**Figure 8:**
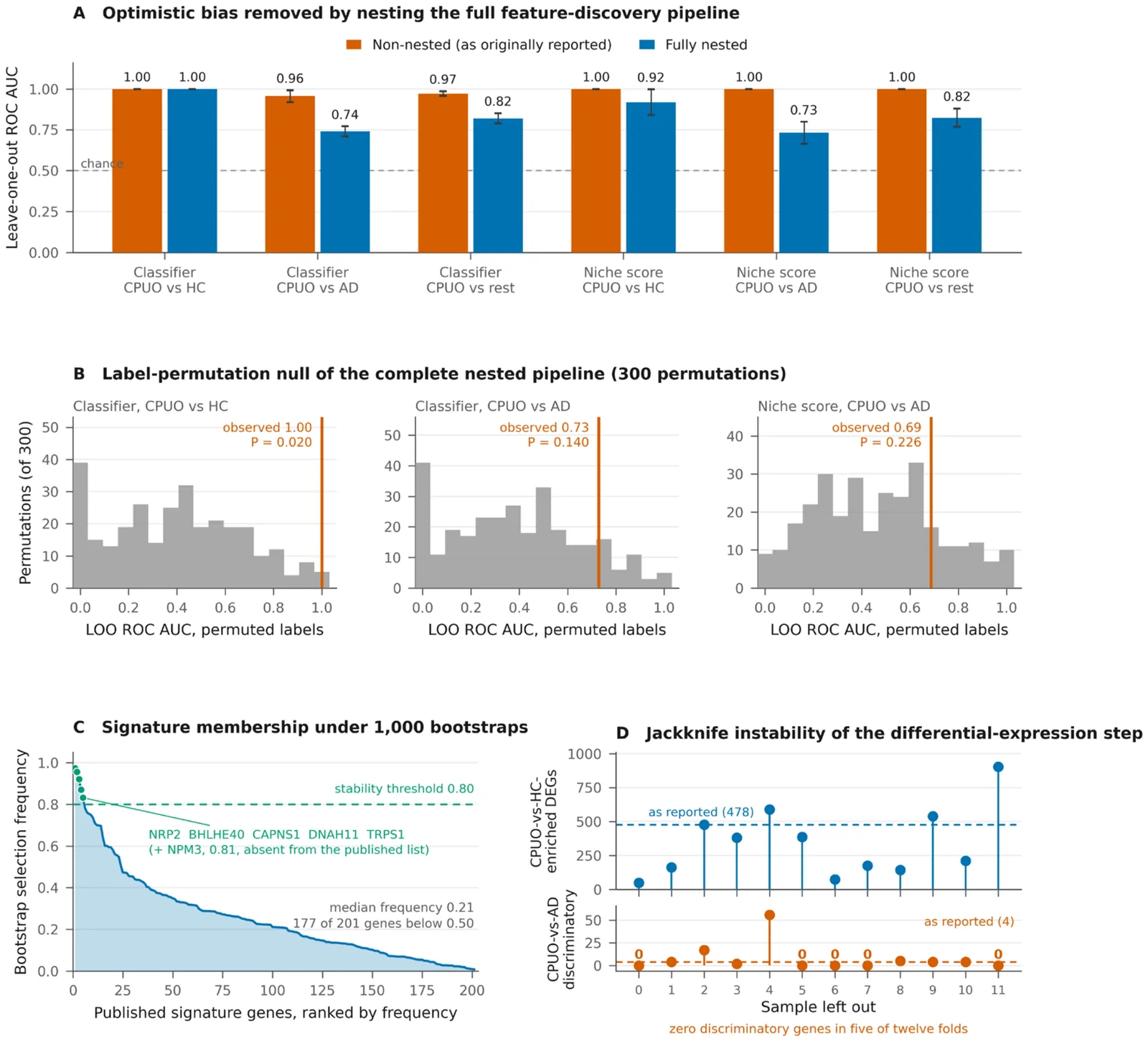
Nested re-validation, label permutation and signature stability. (**A**) Leave-one-out ROC AUC for the latent classifier and for the composite niche score under the non-nested protocol used in the original analysis and under the fully nested protocol, in which differential expression, feature selection, model training, driving-gene derivation, module assignment, z-scaling, and component orientation are all recomputed within each fold. Bars show the mean of 20 repeats at a 700-gene feature budget, and error bars show one standard deviation. (**B**) Empirical null distributions obtained by permuting the group labels 300 times and re-running the complete nested pipeline within each permutation; the vertical line marks the observed value. The null reaches an AUC of 1.00, so perfect separation is attainable under randomized labels. (**C**) Selection frequency of each of the 201 reported signature genes across 1000 stratified bootstrap resamples in which the entire driving-gene chain was re-derived; the dashed line marks the 0.80 stability threshold, and the labeled genes constitute the stable core. (**D**) Number of differentially expressed genes recovered in each of the twelve jackknife subsets, for the CPUO-versus-HC-enriched set (upper) and the CPUO-versus-AD discriminatory set (lower); dashed lines mark the values obtained in the full cohort.

Because every result above was produced in the same twelve samples that defined the signature, we re-executed the complete analysis under the nested protocol described in Section 2.11, using the non-nested protocol as a comparator. Removing the leakage does not affect the CPUO-versus-healthy contrast: leave-one-out AUC for the latent classifier remained 1.000 ± 0.000 across 20 repeats at the 700-gene feature budget and across 10 repeats at the 2000-gene budget. It affects every contrast involving AD. The classifier fell from 0.956 ± 0.036 to 0.741 ± 0.031 for CPUO versus AD, and from 0.972 ± 0.014 to 0.820 ± 0.032 for CPUO versus the two comparator groups combined; the composite niche score fell from 1.000 to 0.919 ± 0.079 against healthy skin, to 0.731 ± 0.068 against AD, and to 0.825 ± 0.056 against both (Figure 8A). The composite score also reversed the ordering of its two contrasts between feature budgets, giving 0.919 against healthy skin and 0.731 against AD at 700 genes but 0.688 and 0.819 respectively at 2000 genes, so it is not stable enough to serve as a stratification tool even within this cohort. All per-protocol classification values reported in this section are consolidated in Table 2. The composite niche score evaluated under the nested protocol is the four-component variant defined in Section 2.9, whereas the value of 1.000 it is compared against is the five-component score of Section 3.8; the two rows therefore differ in component set as well as in protocol. As reported in Section 3.8, the discovery-cohort contrasts are identical for the two variants, so this does not affect the direction of the comparison.

Label permutation of the complete nested pipeline places these values against the appropriate null (Figure 8B; Table 2). Over 300 permutations, the observed CPUO-versus-healthy AUC of 1.00 for the latent classifier was reached or exceeded in 2.0% of permutations (*p* = 0.020) and the corresponding balanced accuracy in 1.7% (*p* = 0.017), and the CPUO-versus-non-CPUO AUC of 0.823 in 4.0% (*p* = 0.040). The CPUO-versus-AD values were not distinguishable from the null for either the classifier (observed 0.729; *p* = 0.140) or the composite score (observed 0.688; *p* = 0.226). We report the complete permutation table rather than a selection of it, because one entry changes what this study can claim: the composite niche score against healthy skin, observed AUC 0.917, was matched or exceeded in 8.6% of permutations (*p* = 0.086) and therefore does not exceed its own null. The corresponding CPUO-versus-non-CPUO value is also non-significant (0.802; *p* = 0.116). The evidence that CPUO skin differs from healthy skin under a fully nested protocol therefore rests on the latent classifier, and not on the composite score, which we accordingly present as a descriptive summary throughout. Three features of the null distributions bear on how the preceding sections should be read. Their maximum was 1.000, and for the composite score against healthy skin even the 95th percentile was 1.000: under randomly assigned labels this pipeline routinely produces perfect leave-one-out separation, so an AUC of 1.00 is not by itself evidence of biological structure. Their means were 0.39 to 0.48, that is slightly below chance; this reflects the known downward bias of leave-one-out AUC estimates at eight to twelve samples rather than an absence of bias, and it does not affect the validity of the permutation test, because the observed statistic and the null are produced by the identical procedure. When the same permutation procedure was applied to the non-nested protocol over 200 permutations, the null distribution was almost unchanged while the observed CPUO-versus-AD value rose from 0.729 to 0.958; leakage raises performance under the true labels without raising the noise floor, which is the definition of optimistic bias [21,22].

Resampling shows that the gene-level signature is the least reproducible element of the study (Figure 8C,D). Across 1000 stratified bootstrap resamples in which the whole chain was re-derived, the median Jaccard overlap with the reported 201 genes was 0.112, and the median recovery was 23.9%; across the twelve jackknife subsets, the corresponding values were 0.299 and 46.0%. In total, 7149 distinct genes entered the signature in at least one resample. Only 5 of the 201 reported genes were selected in at least 80% of bootstrap resamples, and the six genes meeting that threshold overall were *NRP2* (0.974), *BHLHE40* (0.957), *CAPNS1* (0.922), *DNAH11* (0.871), *TRPS1* (0.832), and *NPM3* (0.808), the last of which is absent from the reported list. Module sizes were correspondingly unstable, the keratinocyte-stress group ranging from 1 to 445 genes and the neuro-sensory group from 1 to 527 genes across resamples, so the 128/58/13/2 partition reported in Section 3.3 describes one resample rather than an established structure. The differential expression step is the origin of much of this instability: leaving out a single sample changed the number of CPUO-versus-HC-enriched genes from 50 to 903, and the number of CPUO-versus-AD discriminatory genes was zero in five of the twelve jackknife folds and 56 in one, against the four obtained in the full cohort (Figure 8D).

We therefore restate the central claim of this study in the form the data support. CPUO skin is transcriptomically distinct from healthy skin: the fully nested latent classifier separates the two completely and is unlikely to do so under randomized labels (permutation *p* = 0.020). That claim is carried by the classifier alone; the composite niche score reaches an AUC of 0.919 ± 0.079 against healthy skin but does not exceed its permutation null (*p* = 0.086), and we therefore do not offer it as statistical evidence for the same claim. CPUO skin is not robustly distinguishable from atopic dermatitis skin at this sample size, and the perfect separation reported for that contrast in Section 3.2 and Section 3.8 is a property of the analysis protocol rather than of the disease. Statements at the level of individual genes and of module structure should be limited to the stable core, and the composite score should be read as a descriptive summary of the discovery cohort rather than as a classifier [29,30].

### 3.10. The Niche Score Is Reduced, Not Elevated, in Independent Pruritic and Lesional Skin Cohorts

Because no second public CPUO skin cohort exists, we could not test transportability to new CPUO patients directly. We instead tested the next most consequential question: whether the niche score behaves as a CPUO-associated quantity or as a generic marker of inflamed or pruritic skin. The 201-gene signature, its module assignments, the curated fibroblast and myeloid marker sets and the five component orientation multipliers were frozen from the discovery cohort, and the score was recomputed without refitting in two independent public series: GSE210854, a prurigo nodularis (PN) cohort with pruritic lesional and paired non-pruritic non-lesional skin from five patients plus five healthy samples [27], and GSE174582, an atopic dermatitis series of four normal, seven uninvolved and 23 involved skin samples spanning acute, subacute and chronic lesions [28]. Both are microarray series on platforms different from the discovery data; because module scores are mean within-sample percentile ranks, they are invariant to platform-specific scaling. Signature coverage was 152 of 201 genes (75.6%) in GSE210854 and 114 of 201 (56.7%) in GSE174582, with the keratinocyte-stress module least well covered in the latter (59 of 128 genes; Supplementary Table S4).

**Figure 9:**
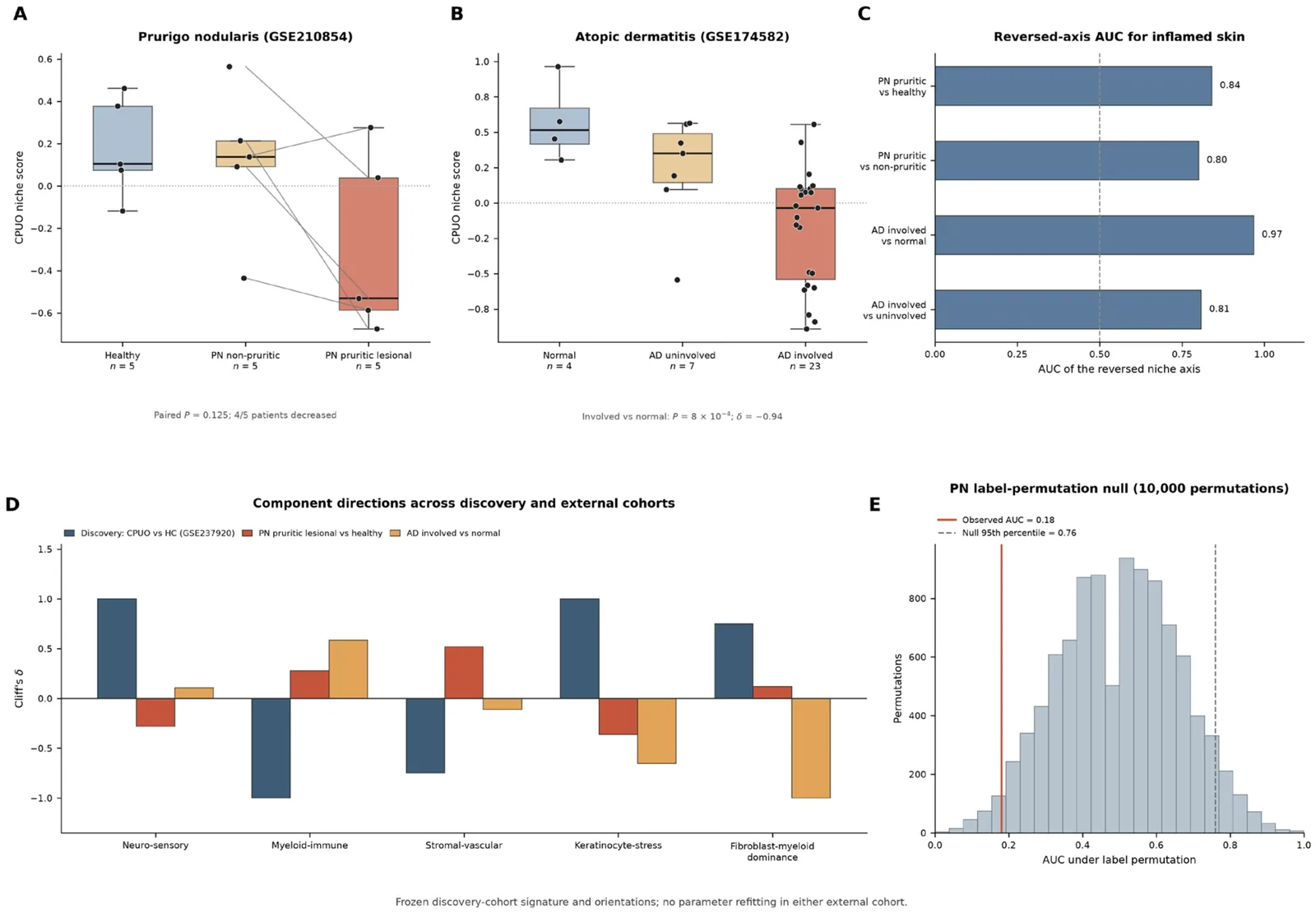
External evaluation of the CPUO neuroimmune-stromal niche score in two independent public skin cohorts. The 201-gene signature, module assignments, curated fibroblast and myeloid marker sets, and five component-orientation multipliers were frozen from the GSE237920 discovery cohort; no parameter was refitted in either external cohort. (**A**) Composite niche scores in GSE210854 prurigo nodularis (PN) samples, comprising healthy skin, non-pruritic non-lesional skin, and pruritic lesional skin. Points represent individual samples, boxes show the median and interquartile range, and grey lines connect the paired non-pruritic and pruritic samples from the same patient. Four of five patients showed a lower score in pruritic lesional skin than in paired non-pruritic skin (two-sided exact Wilcoxon signed-rank *p* = 0.125). The grey dotted horizontal line indicates a niche score of zero. (**B**) Composite niche scores in GSE174582, comprising normal skin and uninvolved and involved atopic dermatitis (AD) skin. Points represent individual samples, and boxes show the median and interquartile range. Involved AD skin had a lower score than normal skin (Cliff’s delta = −0.94; two-sided exact Mann–Whitney *p* = 0.0008). The grey dotted horizontal line indicates a niche score of zero. (**C**) AUC of the sign-reversed niche-score axis for four inflamed-versus-uninflamed contrasts. The grey dashed vertical line indicates the chance-level AUC of 0.50. (**D**) Cliff’s delta for each of the five score components in the discovery CPUO-versus-HC contrast and in the PN-pruritic-lesional-versus-healthy and AD-involved-versus-normal external contrasts. Positive values indicate higher component scores in the first group of each contrast, negative values indicate lower scores, and the black horizontal line indicates Cliff’s delta = 0. (**E**) Null distribution of the composite-score AUC obtained from 10,000 label permutations in the PN cohort. The red solid vertical line marks the observed AUC of 0.18, and the grey dashed vertical line marks the 95th percentile of the permutation-null distribution (AUC = 0.76). These external-cohort analyses evaluate transportability of a frozen score and do not constitute clinical diagnostic validation.

The score was not elevated in either cohort. It was reduced. In GSE210854 the mean score was +0.181 in healthy skin, +0.115 in non-pruritic non-lesional PN skin and −0.295 in pruritic lesional PN skin (lesional versus healthy AUC = 0.16, Cliff’s delta = −0.68, exact Mann–Whitney *p* = 0.095), and the score fell in four of five patients in the within-patient pruritic versus non-pruritic comparison (paired Wilcoxon *p* = 0.125). In GSE174582, the separation was larger and statistically unambiguous: mean +0.575 in normal skin, +0.235 in uninvolved AD skin and −0.171 in involved AD skin (involved versus normal AUC = 0.03, Cliff’s delta = −0.94, exact *p* = 8.0 × 10^−4^; involved versus uninvolved delta = −0.62, *p* = 0.016), with no ordering across acute, subacute, and chronic lesion stages. Reversing the sign of the score yields near-complete discrimination of inflamed from uninflamed skin (AUC 0.84 for PN lesional versus healthy and 0.97 for AD involved versus normal), so the axis itself is reproducible across cohorts and platforms; what does not transfer is its direction (Figure 9A–C). Two of the three pre-specified sensitivity analyses defined in Section 2.10 leave this picture unchanged and one qualifies it. Replacing within-cohort z-scaling by z-scaling referenced to the healthy or normal group of each cohort changes essentially nothing: involved AD skin remains at Cliff’s delta −0.93 against normal skin (exact *p* = 8.0 × 10^−4^) and the prurigo nodularis contrasts are numerically identical, so the majority of lesional samples in GSE174582 are not what produces the reversal. Restricting the discovery cohort to the 114 signature genes measurable on the GPL96 platform likewise leaves CPUO completely separated from healthy skin (delta +1.00, *p* = 0.0286), although the CPUO-versus-AD contrast falls to +0.75 (*p* = 0.114) in that reduced gene set, so the reversal is not a coverage artifact either. The qualification concerns the fibroblast-myeloid dominance component. Removing it strengthens the prurigo nodularis result, where lesional skin then separates from healthy skin at delta −0.84 (*p* = 0.032) and the score falls in five of five patients in the within-patient comparison (paired Wilcoxon *p* = 0.0625), but it substantially weakens the atopic dermatitis result, where involved versus normal falls from delta −0.93 (*p* = 8.0 × 10^−4^) to −0.61 (*p* = 0.059) and involved versus uninvolved from −0.61 (*p* = 0.016) to −0.21 (*p* = 0.43). The reversal in the atopic dermatitis cohort is therefore carried to a substantial degree by the component that counts the myeloid program a second time, whereas the reversal in prurigo nodularis is not, and is in fact obscured by it.

Component-level analysis localizes the reversal (Figure 9D). The myeloid-immune module, which is lower in CPUO than in either comparator group in the discovery cohort (Cliff’s delta −1.00 versus HC), is higher in lesional skin in both validation cohorts (+0.28 in PN, +0.59 in AD); the keratinocyte-stress module, which is higher in CPUO (+1.00), is lower in lesional skin (−0.36 in PN, −0.65 in AD). Only the fibroblast-myeloid dominance component retained its discovery direction in the PN cohort, where it was in fact the strongest single discriminator of pruritic from non-pruritic skin within the same patients (delta +0.76, *p* = 0.056), while reversing in AD (delta −1.00). A 10,000-fold label permutation in the PN cohort placed the observed composite AUC of 0.18 in the lower tail of a null distribution centered on 0.50, indicating that the discordance is systematic rather than a small-sample fluctuation (Figure 9E). The four inflamed-versus-uninflamed contrasts summarized in Figure 9C give sign-reversed AUCs of 0.84 (PN pruritic lesional versus healthy), 0.80 (PN pruritic versus non-pruritic within patients), 0.97 (AD involved versus normal), and 0.81 (AD involved versus uninvolved).

Two readings of this reversal must be separated. Because the composite score was deliberately oriented against the myeloid-immune program in the discovery cohort, part of its reduction in inflamed skin is a built-in consequence of that orientation, and the near-perfect classification of inflamed from uninflamed skin by the sign-reversed axis (AUC 0.84 in prurigo nodularis and 0.97 in atopic dermatitis) shows that the dominant quantity the score captures is cutaneous inflammation status. The informative result is therefore not that the score is unrelated to inflammation—it is strongly and inversely related—but the pole CPUO occupies on that axis. Across three independent cohorts and two microarray platforms, CPUO skin consistently sits at the low-myeloid, high-epidermal-stress end, the mirror image of the pole occupied by pruritic prurigo nodularis lesions and involved atopic dermatitis skin, and this ordering is carried by the two largest and best-covered components of the score (myeloid-immune and keratinocyte-stress). This repositions CPUO skin as a qualitatively low-inflammatory, epidermal-stress-dominated cutaneous state rather than an attenuated form of a classic inflammatory dermatosis—a positive characterization obtained entirely in cohorts independent of the one in which the axis was defined. Two boundaries remain, and we state them explicitly. This is a statement about where CPUO lies relative to inflammatory lesional skin, not a CPUO-specific niche encoded by the score; and it is discriminant validity, not positive validation, because no independent CPUO skin cohort is publicly available and transportability to new CPUO patients is therefore untested. One further discordance must be stated because it is not resolved by our data. In the discovery cohort, the composite score is higher in AD skin than in healthy skin (Cliff’s delta +0.875), whereas in GSE174582 it is markedly lower in involved AD skin than in normal skin (delta −0.94). The same frozen score therefore orders AD relative to healthy skin in opposite directions in the two cohorts. Three explanations are compatible with what we can observe and we cannot adjudicate between them: the four AD samples in GSE237920 may not represent active lesional disease, since the deposited metadata do not record lesion status, disease activity, treatment or biopsy site; the two series use different platforms with different signature coverage; and the discovery cohort contains four samples per group, at which a Cliff’s delta of +0.875 is a single sample-pair away from complete overlap. This has a direct consequence for how the withdrawal of the CPUO-versus-AD claim in Section 3.9 should be read: what the nested analysis shows is that CPUO cannot be separated from the AD samples in this particular cohort, which is a weaker statement than an inability to separate CPUO from atopic dermatitis as a disease.

### 3.11. Summary of the Proposed Computational Model

**Figure 10:**
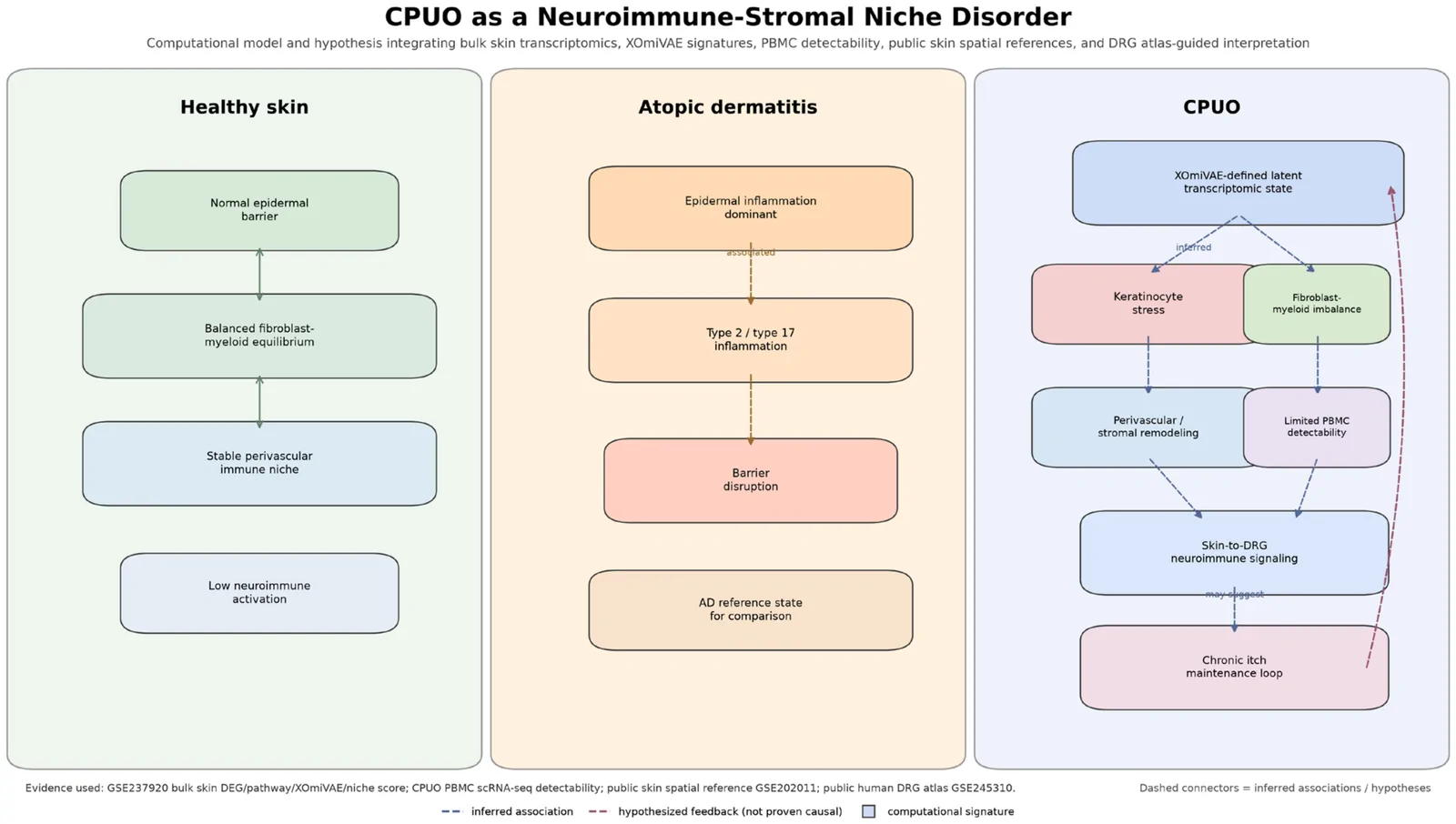
Computational mechanism model. Proposed computational model of CPUO as a neuroimmune-stromal niche disorder. Healthy skin is represented by a stable epidermal barrier, balanced fibroblast and myeloid programs, stable perivascular immune organization, and low neuroimmune activation. AD is represented as an epidermal inflammatory comparator. CPUO is modeled as a latent transcriptomic state involving keratinocyte stress, a directionally consistent but non-significant shift in fibroblast-myeloid dominance, limited stromal-vascular representation, partial PBMC detectability of selected skin-derived signatures, and atlas-guided skin-to-DRG neuroimmune signaling. Dashed connectors denote inferred associations and hypothesized feedback rather than measured relationships; the figure is a hypothesis summary, not a result.

The analyses above can be summarized as a single working model, presented as a hypothesis rather than a result. In this framework, healthy skin is the baseline reference state, with a stable epidermal barrier, balanced fibroblast and myeloid programs, stable perivascular immune organization, and low neuroimmune activation. Atopic dermatitis serves as an inflammatory comparator dominated by epidermal inflammation, a type 2/type 17 immune context, and barrier disruption. CPUO is characterized in our data by a latent transcriptomic state combining keratinocyte stress, a directionally consistent but non-significant shift in fibroblast-myeloid dominance, limited stromal-vascular representation, partial peripheral immune detectability, and atlas-guided links to sensory-neuroimmune signaling. Each element carries the evidential weight established in the corresponding section above, and the connections between them are inferred rather than measured (Figure 10).

Crucially, this proposed mechanism represents a hypothesis-generating computational model rather than definitive causal proof. While PBMC scRNA-seq confirmed the partial peripheral detectability of the CPUO signature, it does not substitute for skin-derived single-cell profiling. Similarly, the spatial and DRG mappings provided reference-guided inferences rather than direct spatial or transcriptional measurements of CPUO patient tissues. These methodological boundaries delineate the limits of our current evidence and highlight clear priorities for future research: external validation in independent skin bulk transcriptomic cohorts, the generation of CPUO-specific single-cell and spatial datasets, and functional testing within mechanistic experimental models (Figure 10).

## 4. Discussion

In this hypothesis-generating computational study, we integrated bulk skin transcriptomics, XOmiVAE-inspired latent modeling, peripheral blood single-cell profiling, and reference-guided spatial and DRG mappings to investigate chronic pruritus of unknown origin (CPUO). The central finding is that CPUO skin carries a latent transcriptomic state that separates it from healthy skin within the evaluated GSE237920 cohort, and that this separation, unlike the apparent separation from atopic dermatitis (AD), survives a fully nested re-analysis (Section 3.9). It is important to be precise about which analysis carries this claim. Conventional differential expression identified broad CPUO-versus-healthy changes but only four CPUO-versus-AD discriminatory genes and no enriched pathway, so at the DEG level, CPUO and AD skin are not separable at this sample size; the separation emerges only in the latent representation and the composite niche score. Pathway-level analyses supported epidermal stress, vascular remodeling, and immune signaling involvement in CPUO. Together, these results are consistent with, but do not by themselves establish, the proposition that CPUO is a distinct cutaneous transcriptional configuration rather than an attenuated inflammatory state [32,33]. We emphasize at the outset that the separation from AD did not survive that re-analysis and is not claimed here.

The defensible contribution of this study is deliberately narrow and twofold. Biologically, CPUO skin occupies a transcriptomic state that the fully nested latent classifier separates from healthy skin against a permutation null, and that lies opposite to classic inflammatory lesional skin along its dominant myeloid-immune and keratinocyte-stress axes—an axis-level, discriminant-validity claim that does not depend on the exact membership of the gene signature, and that does not rest on the composite score, whose separation from healthy skin does not itself survive the same permutation test. Methodologically, we provide a fully worked template in which every label-dependent step of a small-cohort latent-modeling pipeline is re-executed inside cross-validation, subjected to label permutation and bootstrap stability analysis, and reported alongside the original leaked estimates, so that the boundary between what the data support and what the analysis manufactured is made explicit. We do not claim a validated CPUO classifier, a stable individual-gene signature, or distinctness from atopic dermatitis.

The latent model provided a complementary layer of interpretation beyond conventional differential gene ranking. Instead of relying solely on individual DEGs, it identified a compact latent disease state and enabled the prioritization of CPUO-driving genes through interpretable latent dimensions, gene-latent correlations, and encoder-derived contribution estimates. The resulting signature encompassed keratinocyte-stress, myeloid-immune, neuro-sensory, and stromal-vascular modules. This dimensionality reduction approach is particularly advantageous in biologically complex datasets, where disease-associated signals are distributed across coordinated transcriptional programs rather than captured by single markers. However, given the small cohort size, this latent representation remains exploratory pending external validation [34].

The standing of the model family itself warrants direct comment, and we agree that uptake has been minimal. A search for “XOmiVAE” in title, abstract and full text up to August 2026 returns the original report [20] and its associated code repository together with only isolated downstream work; we could not identify an independent study that applies XOmiVAE to a disease outside the cancers used in its development, and to our knowledge the present analysis is the first application of an XOmiVAE-derived approach outside oncology. XOmiVAE therefore cannot be regarded as an established analysis platform, and we make no claim that it is a clinically validated tool. The present work does not in fact use XOmiVAE itself, but an expression-only adaptation that retains its variational encoder–decoder with a supervised head while replacing its DeepSHAP attribution module; the differences are enumerated in Section 2.4 so that our results are not read as evidence about XOmiVAE. What has been adopted is the wider family to which it belongs. Its direct predecessor OmiVAE showed that a variational autoencoder with a supervised head can classify pan-cancer multi-omics profiles [35], and interpretable variational autoencoders in which latent dimensions are tied to defined gene programs, notably VEGA [36] and expiMap [37], are in routine use for single-cell atlas annotation and perturbation modeling, with a recent survey summarizing the field [38]. The clinical position of all of these methods, including ours, is the same. They are hypothesis-generating instruments for prioritizing coordinated transcriptional programs in high-dimensional data, and none has been prospectively evaluated as a diagnostic or prognostic device. Our own results illustrate why that distinction matters, since the apparent classification accuracy of the present adaptation did not survive nested re-validation (Section 3.9). Any clinical use of this class of model would require the evidence demanded of any biomarker: prespecified endpoints, independently collected cohorts, and performance estimated outside the data used for development [29,30]. On present evidence, the appropriate use of XOmiVAE-derived models in dermatology is to generate candidate programs for testing in purpose-collected cohorts, not to classify patients.

A major implication of this analysis is that CPUO pathogenesis may involve the disruption of a neuroimmune-stromal cutaneous niche. The enrichment of pathways related to epidermal differentiation, angiogenesis, and cytokine signaling (for example, MAPK and PI3K-Akt) is consistent with a multicellular neighborhood model in which keratinocyte stress, stromal remodeling, immune activation, and neuro-sensory signaling interact within the skin microenvironment. The public spatial reference located the signature modules within normal skin architecture, but because that reference is a psoriasis dataset and the signature scored highest in psoriatic lesional skin, it establishes only that the signature tracks inflammatory and epidermal-stress activity in skin generally. It does not provide evidence that the spatial organization is CPUO-specific, and the niche interpretation therefore remains a working model rather than a spatially validated finding.

The public GSE295457 peripheral blood/PBMC scRNA-seq analysis provided an important boundary condition for this model, and also required us to reconcile our results with the primary report of that dataset. Several signature genes were detectable in circulating immune compartments, particularly within immune-related modules, indicating that some disease-associated programs may have peripheral immune correlates. In sample-level whole-cell pseudo-bulk analysis, only *FKBP15* remained significant after multiple-testing correction and was lower in CPUO, while signature-overlapping PBMC genes were not FDR-significant and cell-type composition differences did not survive correction. Nominal exploratory overlaps, including *CXCR4*, *BHLHE40* and *CD14*, suggest possible peripheral immune alignment with selected skin-derived programs but require independent validation. This limited aggregate detectability should not be interpreted as a failure of the skin-derived signature; rather, it indicates that important CPUO-associated signals are skin-localized or tissue-resident, and, as set out next, that whole-cell aggregation is itself a substantial part of the explanation.

Our peripheral analysis requires explicit reconciliation with the primary report of the GSE295457 dataset, which identified *CCL3* upregulation in CPUO monocytes and NK cells, showed that plasma CCL3 distinguishes CPUO from healthy controls and correlates with itch severity, and provided evidence that CCL3 sensitizes sensory neurons through CCR1 [25]. Our initial whole-cell pseudo-bulk analysis did not recover this signal, reporting *CCL3* at a log2 fold change of +1.47 with FDR = 0.70, and our earlier framing of a ‘limited peripheral signal’ therefore sat in apparent conflict with a published finding derived from the same data. Two features of our analysis account for the discrepancy. First, aggregating all QC-retained cells per sample averages a monocyte-restricted transcript across a population in which NK and T cells together constitute roughly two-thirds of the cells, which approximately halved the observed effect. Second, our significance rule required both FDR < 0.05 and |log2 fold change| > 0.5 across four samples per group, a conjunction that no gene other than *FKBP15* satisfied. When we repeated the comparison within cell types, *CCL3* was elevated in CPUO in every myeloid compartment, with the largest effects in non-classical (+2.92) and classical (+2.43) monocytes and a concordant direction in NK cells. Our data are therefore consistent with, and provide independent methodological support for, the published *CCL3* finding; the limited peripheral signal we originally reported is a property of whole-cell aggregation and should not be generalized. We have accordingly added *CCL3-CCR1* to the candidate skin-to-DRG axes evaluated in the DRG atlas. Two boundaries on this agreement deserve statement. *CCL3* was not nominated by the skin-derived signature, which is expected given that the signature was learned from bulk skin rather than blood, but it does mean this study offers no cutaneous corroboration of the CCL3 axis. And the reciprocal reduction in *CCR1* we observed in CPUO myeloid cells, while compatible with ligand-driven receptor downregulation, is nominal, unreplicated, and not addressed by the original report.

One internal tension deserves direct treatment. In skin, the inferred myeloid marker program and the myeloid-immune signature module were both lower in CPUO than in either comparator group (Cliff’s delta −1.00 versus HC and versus AD), whereas in blood the same patients’ monocytes showed elevated *CCL3*. These observations are not contradictory, for three reasons. First, circulating monocyte activation state and tissue myeloid abundance are distinct quantities that need not move together: classical monocytes have a half-life of roughly a day and are continuously mobilized, differentiated, and cleared, so an activated peripheral pool can coexist with unchanged or reduced steady-state myeloid representation in a given tissue [39,40]. Second, our cutaneous myeloid measurement is a marker-set score on bulk skin, a tissue whose transcriptome is dominated by keratinocytes; deconvolution of sparse populations from bulk profiles is unreliable at this cohort size, and a lower marker score is at least as consistent with altered myeloid composition or with dilution by an expanded keratinocyte-stress program as with genuine myeloid depletion [41]. Third, in long-standing inflammation, a reduced tissue myeloid signal may itself be a compensatory or counter-regulatory feature rather than evidence of an inactive niche, since sustained chemokine exposure drives receptor downregulation and altered recruitment dynamics—a reading consistent with the reciprocal reduction in *CCR1* we observed in CPUO monocytes. We emphasize that all three explanations are post hoc and that our data cannot adjudicate between them: resolving whether cutaneous myeloid cells are fewer, differently polarized, or simply poorly resolved by bulk profiling requires CPUO skin single-cell data with paired blood, which does not currently exist.

The auxiliary SHAP sensitivity analysis returned zero overlap with the 201-gene signature at every rank cutoff and across all three surrogate model families, and we treat this as a substantive limitation rather than an incidental negative. Three considerations bear on its interpretation, none of which fully rescues the result. Attribution is not directly comparable across model families: SHAP values from a random forest or a linear SVM quantify a gene’s marginal contribution to that model’s decision function, whereas our contribution score combines encoder-to-latent weights, gene-latent correlation, and CPUO effect size, so the two rankings answer different questions and need not converge. At eight to twelve samples, surrogate classifiers are also highly unstable, and their top-ranked features can change substantially with the removal of a single sample. Finally, the 201-gene set is not a pure ranking product: 199 of its members were required to be CPUO-versus-HC-enriched DEGs, whereas the surrogate classifiers could draw on any of the 2000 input genes, so non-overlap is arithmetically easier to obtain than it first appears. Even granting all three, zero overlap across four cutoffs and three model families is a stronger negative than these arguments comfortably absorb. We therefore regard the individual gene-level nominations as the least robust element of this study. The module-level scores and the composite niche score, which depend on aggregate expression across dozens to hundreds of genes rather than on the exact membership of the list, are correspondingly more secure than the gene rankings, and readers should weight them accordingly.

A further question is whether our CPUO skin data support the TH2-skewed cutaneous profile and the IL-31 involvement previously reported in chronic itch of unknown origin [5,6]. The answer is partial. The type 2 master transcription factor *GATA3* was significantly upregulated in CPUO versus healthy skin (log2 fold change +1.79, adjusted *p* = 0.032), which is consistent with the earlier immunohistochemical description of an enhanced TH2 skin immune profile. Beyond this, however, support is weak. The canonical type 2 effector cytokines *IL4*, *IL5*, *IL13*, and *IL31* were not represented on the GSE237920 platform after filtering and could not be assessed at all; *IL31RA* (log2 fold change −0.58, adjusted *p* = 0.58) and *OSMR* (−0.17, adjusted *p* = 0.73) were unchanged; and *CCL17* and *CCL22* did not reach significance. *IL33* was nominally reduced (log2 fold change −1.04, *p* = 0.0074) but did not survive correction. The cutaneous immune signal we did observe was myeloid and chemokine-oriented, with *CD14*, *CXCL1* and *CXCR4* among the highest-scoring immune genes, and the inferred myeloid marker program was lower rather than higher in CPUO skin. We also emphasize that the *IL31RA* detection rate reported above (36.4% of nociceptors) comes from the public DRG reference atlas and describes where the receptor is expressed in developing sensory ganglia generally; it is not a measurement in CPUO patients and carries no information about IL-31 pathway activity in this cohort. Our data therefore neither confirm nor exclude a TH2/IL-31 contribution to CPUO. Resolving this will require larger CPUO skin cohorts with protein-level measurement, since the earlier TH2 evidence was immunohistochemical and the IL-31 evidence serological, and neither modality is well recovered by bulk skin transcriptomics at this sample size. The most recent CPUO-specific evidence has come from blood rather than from skin, and it is compatible with either reading of our result: systemic immune profiling reports elevated MRGPRX2 with a distinct inflammatory signature [9], plasma metabolomics identifies a circulating biomarker signature [10], and the 2026 systematic review characterizes the aggregate picture as multifactorial, with type 2 dysregulation as one component alongside neurogenic and aging-related mechanisms rather than as the whole of it [7]. That our cutaneous myeloid program is reduced while these systemic markers are elevated is the same skin-blood dissociation discussed above, and it is a reason not to assume that a peripheral type 2 or mast-cell signature has a cutaneous counterpart.

The inferred fibroblast-myeloid relationship adds another potential mechanistic layer, though it is the weakest statistically supported element of the model. Fibroblasts and monocyte-macrophage lineages are increasingly recognized as reciprocal regulators of tissue repair and chronic inflammatory remodeling [19,42]; the most direct evidence that this balance governs skin integrity comes from mouse genetic models [18] and has not been established for human skin homeostasis, so we invoke it as an organizing concept rather than as established human biology. In our bulk dataset, fibroblast and myeloid marker programs were positively correlated across samples, and the derived fibroblast-myeloid dominance score shifted toward fibroblast predominance in CPUO with a moderate effect size, but no group comparison reached significance, and the component did not survive multiple-testing correction within the composite score. Evaluated ligand-receptor axes—including *CSF1-CSF1R*, *IL34-CSF1R*, *CCL2-CCR2*, CXCL12*-CXCR4*, and *TGFB1-TGFBR1/2*—provide plausible, testable communication networks for future spatial validation, but on present evidence the fibroblast-myeloid axis is a hypothesis carried by the wider literature rather than by our data.

Integration with a public human DRG atlas placed the skin-derived signature in a neuroimmune context relevant to chronic itch [43]. Signature modules mapped onto the atlas, and established itch-related genes (*SCN10A*, *SCN11A*, *NTRK1*, *NTRK2*, *IL31RA*, *OSMR*, and *HRH1*) displayed the expected cell-type-specific distributions across sensory neuron, nociceptor, glial and macrophage compartments, which supports the technical validity of the projection. Its biological reach is nonetheless narrow. The atlas profiles human embryonic DRG from gestational weeks 7–21 [26], so it describes where itch-related receptors are expressed during sensory ganglion development, not their regulation in adult ganglia of patients with chronic itch; receptor repertoires, glial maturation and resident macrophage populations all change substantially after this window. These distributions therefore support the biological plausibility of skin-to-neuron signaling as a class of hypothesis and generate specific targets for future paired skin-neural studies in adult tissue, but they cannot be read as CPUO-associated DRG changes [12]. How this class of inference should be framed has itself moved on. Current reviews of itch neuroimmunology place peripheral neuron-immune signaling and central glial amplification within a single circuit [14,15], and the demonstration that type 2 cytokines alter sensory nerve architecture rather than only nerve activity [16] supplies a concrete mechanism by which a sustained cutaneous cytokine environment could reshape the very DRG populations onto which we projected our signature. Testing that in CPUO would require adult human ganglia, which no public resource currently provides.

The CPUO neuroimmune-stromal niche score offers a quantitative summary of the latent disease state. In this cohort, the composite score separated CPUO completely from both AD and healthy skin by synthesizing neuro-sensory, immune, stromal, epidermal-stress, and fibroblast-myeloid components, and a label-permutation test that repeated the full orientation-and-scoring procedure within each permutation placed the observed separation outside 99.3% of the null distribution. Its value at present lies in disease stratification within a discovery cohort and in prioritizing mechanistic pathways for future work, not in classification performance. Because component orientation was fixed using the same samples in which the score was evaluated, and because both the score and the upstream latent model were developed on 12 samples, the reported ROC and leave-one-out metrics are internal descriptors rather than performance estimates, and validation in larger, clinically diverse and independently collected populations is required before any diagnostic interpretation is warranted. Under the nested protocol, separation is reduced against healthy skin (AUC 0.919 ± 0.079) and lost against AD (0.731 ± 0.068, permutation *p* = 0.226); and against healthy skin, the nested value does not exceed its own permutation null either (*p* = 0.086). The composite score is therefore best described as a compact summary of the discovery cohort and of the axis identified in Section 3.10, and not as evidence in its own right that CPUO skin differs from healthy skin—that evidence comes from the latent classifier.

The nested re-validation reported in Section 3.9 changes what this study can claim, and we prefer to state that plainly rather than to present the original figures with a caveat attached. Three steps used the labels of all twelve samples before cross-validation, and their combined effect was to raise the CPUO-versus-AD leave-one-out AUC from 0.74 to between 0.96 and 1.00 while leaving the permutation null essentially unchanged. The CPUO-versus-healthy contrast is unaffected for the latent classifier and remains significant against a fully nested null; the CPUO-versus-AD contrast, which is the one that would establish CPUO as a state distinct from atopic dermatitis, does not. Reporting the full permutation table rather than a subset of it also cost us the composite score as an independent line of statistical support for the CPUO-versus-healthy claim, and we consider that the correct outcome of the exercise rather than an inconvenience. That is consistent with the differential expression result, in which only four genes separated the two groups, and with the jackknife finding that this number falls to zero in five of twelve folds. We therefore no longer claim that CPUO is transcriptomically distinguishable from AD. We claim that it differs from healthy skin, that the axis along which it differs behaves opposite to lesional inflammatory skin in two independent cohorts, and that establishing distinctness from AD requires a larger and clinically annotated cohort. Reporting the leaked and nested analyses side by side is, in our view, the more durable contribution of this work for transcriptomic studies constrained to comparable sample sizes [21,22,29,30].

The external evaluation clarified what the niche score measures, and it did so in an informative direction. A score oriented against the cutaneous myeloid program is, by construction, an inverse index of inflammatory tone, and consistent with this, the sign-reversed axis separated inflamed from uninflamed skin almost perfectly (AUC 0.84–0.97); we therefore do not claim that the score is unrelated to inflammation. What is not a construction artifact is the pole at which CPUO sits. In the discovery cohort, in prurigo nodularis and in atopic dermatitis, the myeloid-immune module is high in classic lesional skin but low in CPUO, while the keratinocyte-stress module shows the opposite pattern, so CPUO skin occupies the low-inflammatory, high-epidermal-stress end of an axis whose other end is defined by active inflammatory lesions. Read together with the skin-blood dissociation discussed above—reduced cutaneous myeloid signal in CPUO alongside activated peripheral monocytes—this is positive evidence that CPUO is not merely a milder inflammatory dermatosis but a cutaneous state of a different character, in which epidermal stress predominates over local myeloid inflammation. This does not reinstate a claim of transcriptomic distinctness from atopic dermatitis at the classifier level, which the nested analysis does not support (Section 3.9); it is a statement about the inflammatory-versus-epidermal-stress axis, bounded in two ways—it concerns CPUO’s position relative to inflammatory lesional skin rather than a CPUO-specific spatial niche, and because no independent CPUO cohort exists it remains discriminant validity rather than transportability.

### 4.1. Limitations

Several limitations warrant consideration, and we state the methodological ones first because they bear directly on how the results above should be weighted. First, the score and the latent model were developed and evaluated in the same 12 samples, and three steps used the labels of all twelve before cross-validation; under the fully nested protocol, the CPUO-versus-AD separation is not distinguishable from a permutation null (Section 3.9), and all classification performance reported for that contrast should be regarded as unsupported. Second, genes described here as CPUO-versus-HC-enriched were defined as significant in CPUO versus HC and not significant in AD versus HC, which is a difference in significance and not a significant difference [24]; the direct CPUO-versus-AD contrast yielded four genes, and that number falls to zero when any of five individual samples is removed. Third, the auxiliary SHAP sensitivity analysis showed zero overlap with the 201-gene signature across all cutoffs and model families, and bootstrap resampling recovered a median of 24% of the signature, so the gene-level nominations are the least robust component of this study and interpretation should be confined to the six-gene stable core. Fourth, the module gene counts are severely unbalanced—128 keratinocyte-stress genes against 58 myeloid-immune, 13 neuro-sensory, and two stromal-vascular genes—so the composite score is dominated by the keratinocyte-stress program; the two-gene stromal-vascular component should not be regarded as a module, and module sizes were themselves unstable under resampling; module-level tests were additionally not corrected across the four comparisons. Fifth, only 20 of the 201 signature genes were assigned to modules by curated keyword rules, the remaining 181 being assigned by correlation within these same 12 samples, so the module structure is data-dependent and post hoc. Sixth, age, sex, disease duration, itch severity, treatment history, and biopsy site are not resolvable in the GSE237920 metadata; because biopsy site is a major determinant of keratinization-related gene expression and our largest module consists of 128 keratinocyte-stress genes, residual site heterogeneity is a plausible contributor to the group differences we report and cannot be excluded at four samples per group. Seventh, 51 of the 201 signature entries are pseudogenes, lncRNA clone identifiers, or other non-coding features whose high ranking in small bulk cohorts commonly reflects alignment or probe artifacts. Eighth, the limited sample size of GSE237920 constrains statistical power throughout; the *p*-values for the principal comparisons sit at the theoretical floors of their tests, and confidence intervals for the main effect sizes are either degenerate at the boundary or wide enough to include the null (Section 3.8). Ninth, the absence of patient-matched CPUO single-cell or spatial transcriptomics necessitated reliance on public reference atlases and peripheral blood data, and those atlases are imperfect proxies in specific ways: the spatial reference is a psoriasis dataset and the DRG atlas is embryonic rather than adult tissue, while the peripheral *CCL3* result is a reanalysis of the dataset in which that association was originally reported and is therefore not independent of it. Tenth, the external evaluation reported here tests discriminant validity against other dermatoses rather than transportability: it shows that the score is not a generic inflammation or itch marker, but it cannot substitute for an independent CPUO cohort, which does not currently exist in the public domain. Finally, atlas-guided inference cannot establish causal tissue remodeling, and future validation must account for disease heterogeneity, biopsy site and treatment history, all of which could influence signature reproducibility. Three further limitations arise from the analyses added in this revision. First, the composite niche score does not exceed its fully nested permutation null even against healthy skin (observed AUC 0.917, *p* = 0.086), and the 95th percentile of that null is 1.000; the score should therefore be read as descriptive, and the CPUO-versus-healthy claim rests on the latent classifier alone. Second, two of the five components of the composite score carry myeloid information and are both oriented against it, and removing the fibroblast-myeloid dominance component substantially weakens the reversal observed in the atopic dermatitis validation cohort (Section 3.10), so that part of the external result is partly a consequence of how the score is built. Third, the composite score is higher in AD than in healthy skin in the discovery cohort but lower in involved AD than in normal skin in GSE174582; because the GSE237920 metadata do not record lesion status or disease activity, we cannot establish whether the AD samples in the discovery cohort represent active lesional disease, and the inability to separate CPUO from AD reported here is strictly an inability to separate CPUO from the AD samples of that cohort.

### 4.2. Future Directions

Future studies must rigorously test this proposed model using CPUO-specific single-cell sequencing and spatial transcriptomics paired with comprehensive clinical annotation. Spatial co-localization of epidermal stress programs, nerve fibers, myeloid cells, and fibroblast states will be particularly informative. Mechanistic experiments—such as keratinocyte–fibroblast–myeloid–neuron co-cultures or organotypic skin models—are required to evaluate whether the inferred ligand-receptor axes actively drive neuronal activation, and the CCL3-CCR1 axis is a specific priority given the peripheral evidence discussed above. Ultimately, correlating the CPUO niche state with clinical itch intensity, chronicity, and treatment response will determine its translational utility [44]. The current study establishes a computational framework, conceptually positioning CPUO as a neuroimmune-stromal niche disorder and providing clear, testable hypotheses for future mechanistic exploration [45]. The therapeutic landscape has also moved, and it offers a route to the paired tissue this field lacks. Interleukin-31 receptor blockade with nemolizumab, licensed in prurigo nodularis and atopic dermatitis, is being extended to other pruritic indications and the emerging evidence has recently been collated [46]; dupilumab has been evaluated in a dedicated phase 3 CPUO program (LIBERTY-CPUO-CHIC, NCT05263206), whose primary itch endpoint was not met against a high placebo response although secondary itch measures favored active treatment, and which has so far been reported only in conference form. Trials of this kind generate what a computational framework of this sort needs and cannot otherwise obtain: CPUO skin sampled before and after a mechanistically defined intervention, with itch intensity recorded alongside. Because the scoring procedure reported here is frozen and requires no refitting (Section 2.10), it can be applied to such biopsies without further methodological development. We note that as of August 2026, GSE237920 remains, to our knowledge, the only public CPUO skin transcriptomic dataset, so the limitation stated in Section 4.1 is unchanged rather than superseded [7,8].

## 5. Conclusions

This computational study defines an interpretable, XOmiVAE-inspired latent transcriptomic state in chronic pruritus of unknown origin that spans keratinocyte-stress, myeloid-immune, neuro-sensory, and stromal-vascular gene groups and that separates CPUO from healthy skin within a twelve-sample discovery cohort. That separation survives a fully nested re-analysis in which every label-dependent step is recomputed within each cross-validation fold, and it is unlikely to arise under randomized labels (permutation *p* = 0.020). This surviving claim is carried by the latent classifier; the composite niche score derived from the same signature reaches an area under the curve of 0.919 ± 0.079 against healthy skin but does not exceed its own permutation null (*p* = 0.086) and is reported here as a descriptive summary rather than as evidence. The corresponding separation from atopic dermatitis does not survive: it falls from an area under the curve of 1.00 to 0.74 under the nested protocol and is not distinguishable from the permutation null, so we do not claim that CPUO is transcriptomically distinct from atopic dermatitis at this sample size. Cell-type-restricted analysis of public peripheral blood single-cell data reproduces the previously reported CPUO-associated *CCL3* upregulation in monocytes that whole-cell aggregation obscures, and atlas-guided projection onto public skin spatial and human dorsal root ganglion references places the signature in a plausible but unvalidated neuroimmune-stromal context.

Frozen application of the composite score to two independent skin cohorts repositions CPUO along a cutaneous inflammation-versus-epidermal-stress axis: pruritic prurigo nodularis lesions and involved atopic dermatitis skin occupy the high-inflammatory pole, whereas CPUO occupies the low-inflammatory, epidermal-stress-dominated pole, the reversal being carried by the myeloid-immune and keratinocyte-stress components. Because the score was oriented against the myeloid program, this axis is by construction an inverse inflammation index; the finding is the consistent direction CPUO takes on it across three independent cohorts, not a CPUO-specific niche, and it is discriminant validity rather than positive validation in CPUO. The study remains hypothesis-generating, and its own re-validation defines the limits of what it supports.

## Figures and Tables

**Table 1:** Representative CPUO-driving genes nominated by latent feature attribution.

Gene	Module	Driving Score	Gene Contribution	Evidence	Bootstrap Frequency	Function
***BHLHE40***	keratinocyte-stress module	1.900	9.399	2	0.957	Basic helix-loop-helix transcriptional repressor acting downstream of hypoxic, circadian and immune signaling; regulates keratinocyte and T-cell responses
***NRP2***	neuro-sensory module	1.891	6.012	2	0.974	Neuropilin-2; co-receptor for class-3 semaphorins in axon guidance and for VEGF-C in lymphangiogenesis
*CD14*	myeloid-immune module	1.818	4.263	2	0.097	Co-receptor with TLR4 for lipopolysaccharide; monocyte and macrophage lineage marker
*TXNRD1*	keratinocyte-stress module	1.782	3.993	2	0.009	Thioredoxin reductase 1; selenoprotein maintaining the thioredoxin system and cellular redox balance under oxidative stress
*CXCL1*	myeloid-immune module	1.724	3.628	2	0.110	CXC chemokine ligand 1; neutrophil chemoattractant acting through CXCR2, secreted by keratinocytes and fibroblasts
*NRP1*	neuro-sensory module	1.660	3.338	2	0.021	Neuropilin-1; semaphorin-3A co-receptor with plexin A in axon guidance and VEGF-A co-receptor on endothelium
*CXCR4*	myeloid-immune module	1.639	3.255	2	0.031	Receptor for CXCL12; regulates leukocyte trafficking and is expressed by myeloid cells and sensory neurons
*NTRK2*	neuro-sensory module	1.608	3.120	2	0.058	Tropomyosin receptor kinase B; high-affinity receptor for BDNF and neurotrophin-4, mediating sensory neuron survival and sensitization
*THBS1*	stromal-vascular module	1.547	2.827	2	0.044	Thrombospondin-1; matricellular glycoprotein that activates latent TGF-beta and mediates fibroblast-macrophage crosstalk
*MCAM*	stromal-vascular module	1.359	2.196	2	0.008	Melanoma cell adhesion molecule (CD146); adhesion receptor of pericytes, vascular smooth muscle and endothelium, and the second member of the two-gene stromal-vascular component

Representative genes are ordered by CPUO-driving score; the gene-level contribution column reports the raw post hoc attribution weight from which that score was derived. The “Evidence” column is a count, not a score. It records how many of three pre-specified evidence categories support a gene: (i) membership in the CPUO-versus-HC-enriched differentially expressed gene set, (ii) membership in the four-gene CPUO-versus-AD discriminatory set, and (iii) membership in a curated niche pathway gene set. The possible range is therefore 0 to 3. Across the full 201-gene signature, the observed range was 1 to 2, with 190 genes supported by one category and 11 by two. A value of 2 in this table therefore means that the gene met two of the three categories; every gene shown is such a gene, and no gene met all three. The “Function” column gives the established molecular function of each gene product. The “Bootstrap frequency” column gives the proportion of 1000 stratified bootstrap resamples in which the gene was re-selected when the entire derivation chain was repeated (Section 3.9); values at or above 0.80 define the stable core, and only *NRP2* and *BHLHE40* reach it among the genes shown. All eleven genes with two supporting categories are shown, including *MCAM*, the second member of the two-gene stromal-vascular component, with the single exception of the pseudogene *PHF2P2*, which is excluded for the reason given in the text. Gene names are in bold for the two genes that reach the 0.80 bootstrap stability threshold. Readers should note that seven of the ten genes listed have bootstrap frequencies below 0.12, so this table ranks by attribution score and not by reproducibility. The complete 201-gene signature with module assignment, assignment basis, and assignment confidence is provided as Supplementary Table S2, and the complete per-gene bootstrap selection frequencies are provided as Supplementary Table S5. BDNF, brain-derived neurotrophic factor; CXC, C-X-C motif; CXCL, C-X-C motif chemokine ligand; CXCR, C-X-C motif chemokine receptor; TGF, transforming growth factor; TLR4, Toll-like receptor 4; VEGF, vascular endothelial growth factor.

**Table 2:** Consolidated leave-one-out classification performance and label-permutation results under the non-nested and fully nested protocols, at the 700-gene feature budget unless stated. ‘AUC (mean ± SD)’ is the leave-one-out estimate across repeats (20 repeats at 700 genes, 10 at 2000; the non-nested niche score was run once), whereas ‘Permutation observed’ is the single observed statistic entering the label-permutation test; these are different quantities, which is why the two columns do not coincide. Permutation *p*-values for the nested protocol come from 300 permutations and those for the non-nested protocol from 200, in both cases drawn from the 34,650 distinct 4/4/4 label assignments; *p* = (1 + k)/(N + 1). Dashes denote combinations for which no permutation was run. Every permutation result that was computed is listed here. Note that the composite niche score does not exceed its permutation null against healthy skin (*p* = 0.086), and that the reversal of the niche-score ordering between the 700- and 2000-gene feature budgets (last two rows) indicates that the score is not stable enough to serve as a stratification tool even within this cohort.

Contrast	Quantity	Protocol	Feature Budget	AUC (Mean ± SD)	Permutation Observed	Permutation *p*
CPUO vs. HC	Latent classifier	Non-nested	700	1.000 ± 0.000	1.000	0.030
CPUO vs. HC	Latent classifier	Nested	700	1.000 ± 0.000	1.000	0.020
CPUO vs. AD	Latent classifier	Non-nested	700	0.956 ± 0.036	0.958	0.025
CPUO vs. AD	Latent classifier	Nested	700	0.741 ± 0.031	0.729	0.140
CPUO vs. non-CPUO	Latent classifier	Non-nested	700	0.972 ± 0.014	0.969	0.005
CPUO vs. non-CPUO	Latent classifier	Nested	700	0.820 ± 0.032	0.823	0.040
CPUO vs. HC	Niche score	Non-nested	700	1.000 (single repeat)	—	—
CPUO vs. HC	Niche score	Nested	700	0.919 ± 0.079	0.917	0.086
CPUO vs. AD	Niche score	Non-nested	700	1.000 (single repeat)	—	—
CPUO vs. AD	Niche score	Nested	700	0.731 ± 0.068	0.688	0.226
CPUO vs. non-CPUO	Niche score	Nested	700	0.825 ± 0.056	0.802	0.116
CPUO vs. HC	Latent classifier	Nested	2000	1.000 ± 0.000	—	—
CPUO vs. AD	Latent classifier	Nested	2000	0.750 ± 0.072	—	—
CPUO vs. HC	Niche score	Nested	2000	0.688 ± 0.093	—	—
CPUO vs. AD	Niche score	Nested	2000	0.819 ± 0.080	—	—

Abbreviations: AD, atopic dermatitis; AUC, area under the receiver operating characteristic curve; CPUO, chronic pruritus of unknown origin; HC, healthy control; SD, standard deviation.

## Data Availability

All datasets analyzed in this study are publicly available in the Gene Expression Omnibus (GEO). Bulk skin transcriptomes: GSE237920 [23]. Peripheral blood mononuclear cell single-cell RNA sequencing: GSE295457. Skin spatial transcriptomic reference: GSE202011. Human dorsal root ganglion single-nucleus reference: GSE245310. External evaluation cohorts: GSE210854 [27] and GSE174582 [28]. No new data were generated. All project-specific analysis scripts, computational workflows, and custom adapters, including the external evaluation pipeline, are available at https://github.com/zouminn9-oss/CPUO-Neuroimmune-Niche (accessed on 5 August 2026), and will be archived with a Zenodo DOI upon acceptance. The scripts implementing the nested re-validation, the label-permutation test, and the bootstrap stability analysis reported in Section 3.9 (21_nested_cv_validation.py, 22_permutation_test.py, and 23_bootstrap_gene_stability.py) are included in the same repository, together with the NumPy reimplementation and its verification checks.

## Supplementary Materials

The following supporting information can be downloaded at: https://www.mdpi.com/article/10.3390/biomedicines14092000/s1. Figure S1: Bulk expression preprocessing and quality control; Figure S2: Additional differential expression and pathway panels; Figure S3: Exploratory latent model performance; Figure S4: SHAP sensitivity analysis; Figure S5: PBMC scRNA-seq quality control, clustering and markers; Figure S6: Additional PBMC signature module maps; Figure S7: Additional public skin spatial reference panels; Figure S8: Additional fibroblast-myeloid reference evidence; Figure S9: Additional DRG atlas-guided interpretation; Figure S10: Additional CPUO niche score panels; Table S1: Sample-level metadata for the analyzed public series; Table S2: Complete 201-gene CPUO-driving signature; Table S3: Cell-type-restricted PBMC pseudo-bulk differential expression; Table S4: External evaluation of the niche score in GSE210854 and GSE174582 (signature coverage, per-sample scores, group contrasts and component-level effect sizes); Table S5: Nested re-validation, label-permutation and bootstrap stability results (per-protocol performance, permutation null distributions, per-gene bootstrap selection frequencies, module-size distributions and jackknife differential expression counts); Table S6: Gene membership of the custom pathway modules scored by GSVA; Table S7: Curated 16-gene fibroblast and 16-gene myeloid marker sets, with detection status in each cohort; Table S8: Sensitivity analyses (four-component composite score, reference-group standardization, coverage-matched signature and *NRP1*/*NRP2* module reassignment) in the discovery and validation cohorts.

## Abbreviations

AD	atopic dermatitis
AUC	area under the receiver operating characteristic curve
BDNF	brain-derived neurotrophic factor
BH	Benjamini–Hochberg
BP	biological process (Gene Ontology)
CC	cellular component (Gene Ontology)
CI	confidence interval
CPM	counts per million
CPUO	chronic pruritus of unknown origin
CV	cross-validation
DC	dendritic cell
DEG	differentially expressed gene
DRG	dorsal root ganglion
FDR	false discovery rate
GEO	Gene Expression Omnibus
GO	Gene Ontology
GSEA	Gene Set Enrichment Analysis
GSVA	Gene Set Variation Analysis
HC	healthy control
KEGG	Kyoto Encyclopedia of Genes and Genomes
KL	Kullback–Leibler (divergence)
logFC	log2 fold change
LOOCV	leave-one-out cross-validation
MF	molecular function (Gene Ontology)
MSE	mean squared error
NK	natural killer (cell)
PBMC	peripheral blood mononuclear cell
PCA	principal component analysis
PN	prurigo nodularis
QC	quality control
ReLU	rectified linear unit
ROC	receiver operating characteristic
scRNA-seq	single-cell RNA sequencing
SD	standard deviation
SHAP	SHapley Additive exPlanations
snRNA-seq	single-nucleus RNA sequencing
SVA	surrogate variable analysis
SVM	support vector machine
TH2	T helper type 2
UMAP	uniform manifold approximation and projection
VAE	variational autoencoder
