# Supplementary material for: XOmiVAE-Inspired Transcriptomic Analysis of Chronic Pruritus of Unknown Origin: A Cutaneous State Distinct from Healthy Skin but Not Robustly Separable from Atopic Dermatitis
Source: Biomedicines. 2026 Sep 5;14(9):2000. doi: 10.3390/biomedicines14092000 (PMC13604094; doi:10.3390/biomedicines14092000)
Supplement: Supplementary file 1 [file biomedicines-14-02000-s001.zip › Supplementary_Material.pdf]

Supplementary Material

XOmiVAE-Inspired Transcriptomic Analysis of Chronic Pruritus of Unknown Origin: A Cutaneous State Distinct from Healthy Skin but Not Robustly Separable from Atopic Dermatitis

Supplementary Figure S1. Bulk expression preprocessing and QC

(A) Distribution of log2 expression values across the 12 GSE237920 samples after normalization and low-expression filtering. (B) Principal component analysis of the filtered bulk matrix, coloured by group. (C) Sample-sample Pearson correlation heatmap. (D) Hierarchical clustering of samples. Together these panels document that no sample was an outlier and that no batch variable with more than one level was recoverable from the curated metadata.

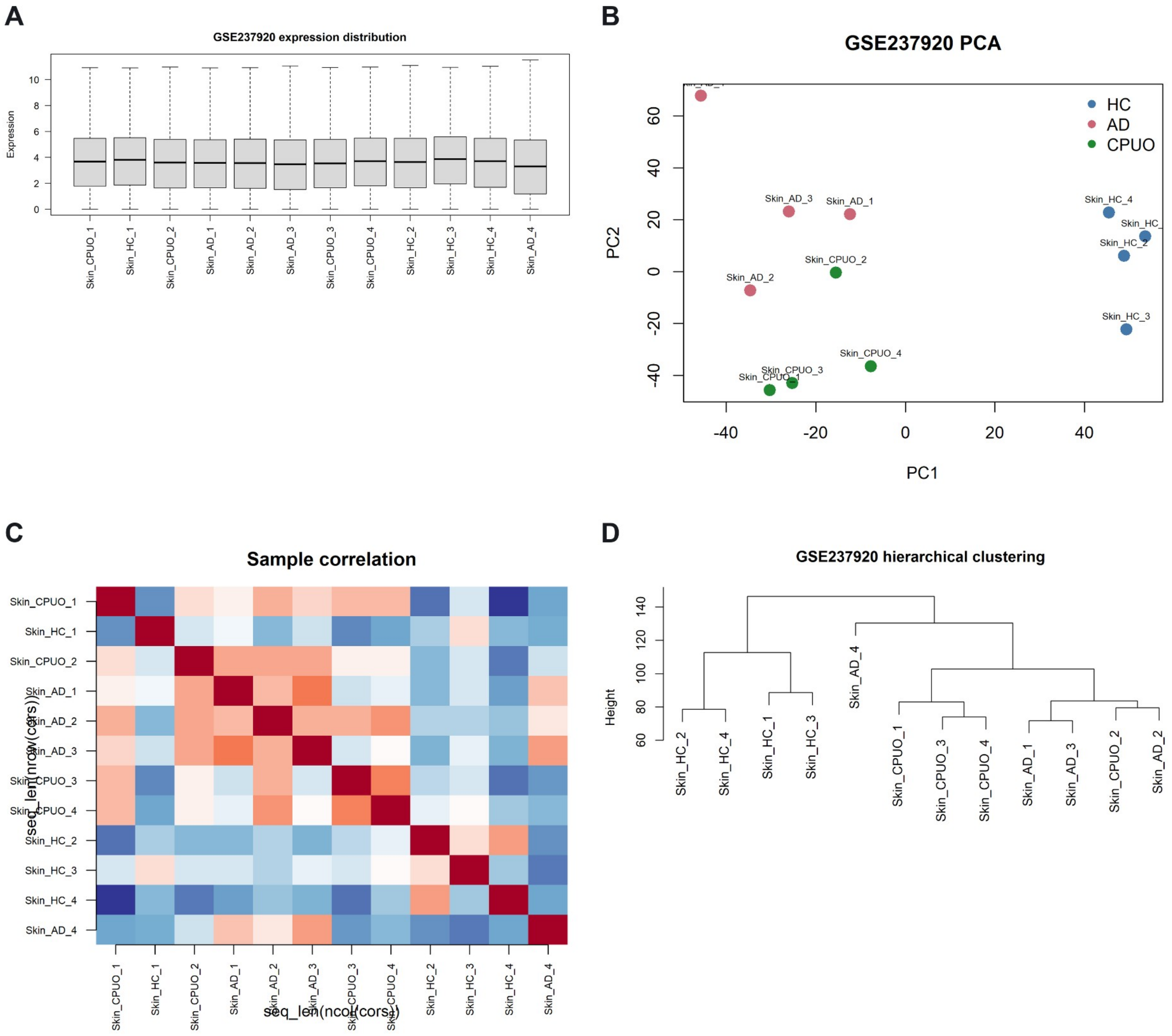

Supplementary Figure S2. Additional DEG and pathway panels

(A) UMAP of the bulk samples by group. (B) Heatmap of the top AD-versus-HC differentially expressed genes. (C) Heatmap of the top CPUO-versus-AD differentially expressed genes, shown for completeness given that only four genes reached the primary threshold. (D) GO biological process dot plot for AD versus HC, illustrating the narrow but highly significant epidermal-differentiation enrichment discussed in the main text. (E) GSEA ridge plot for CPUO versus HC. (F) Pathway activity (GSVA) scores by group.

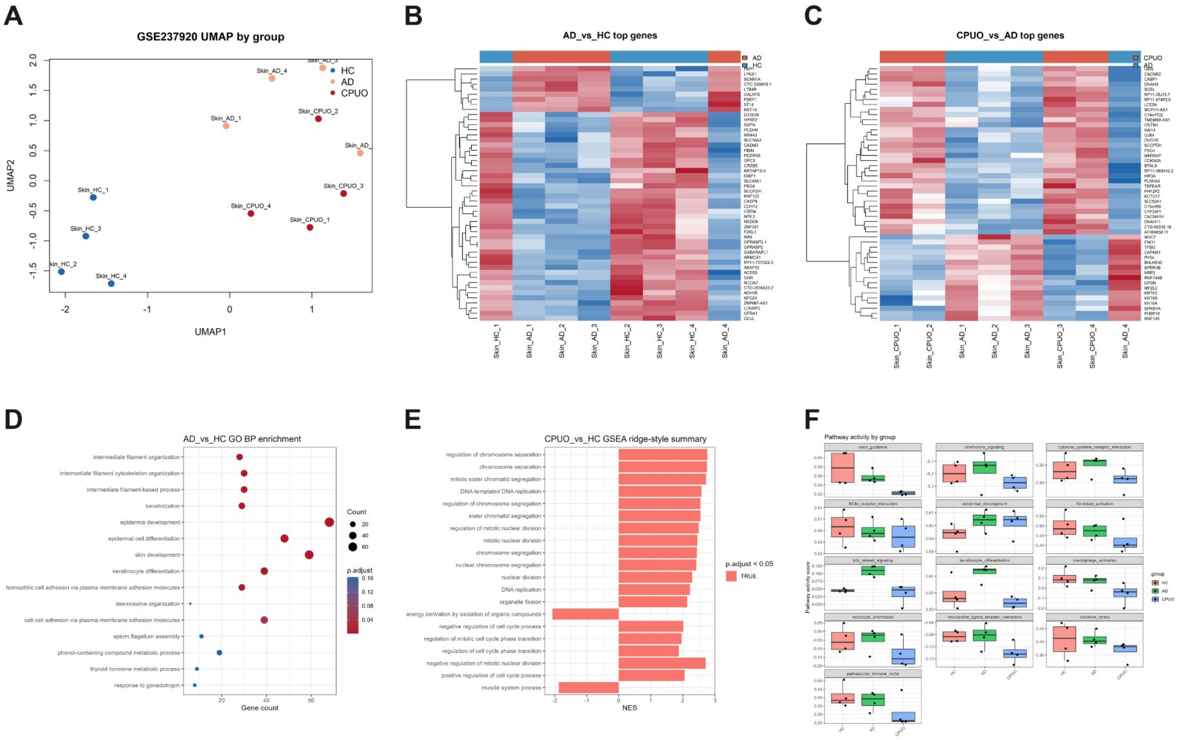

Supplementary Figure S3. XOmiVAE exploratory model performance

(A) Training loss of the XOmiVAE-inspired adaptation. (B) UMAP of the latent representation compared with the input space. (C, D) Exploratory receiver operating characteristic curves for CPUO versus HC and CPUO versus AD. (E, F) Corresponding confusion matrices. All classification metrics derive from 12 samples, are discovery-cohort-internal, and are presented here rather than in the main figures because they cannot estimate generalization.

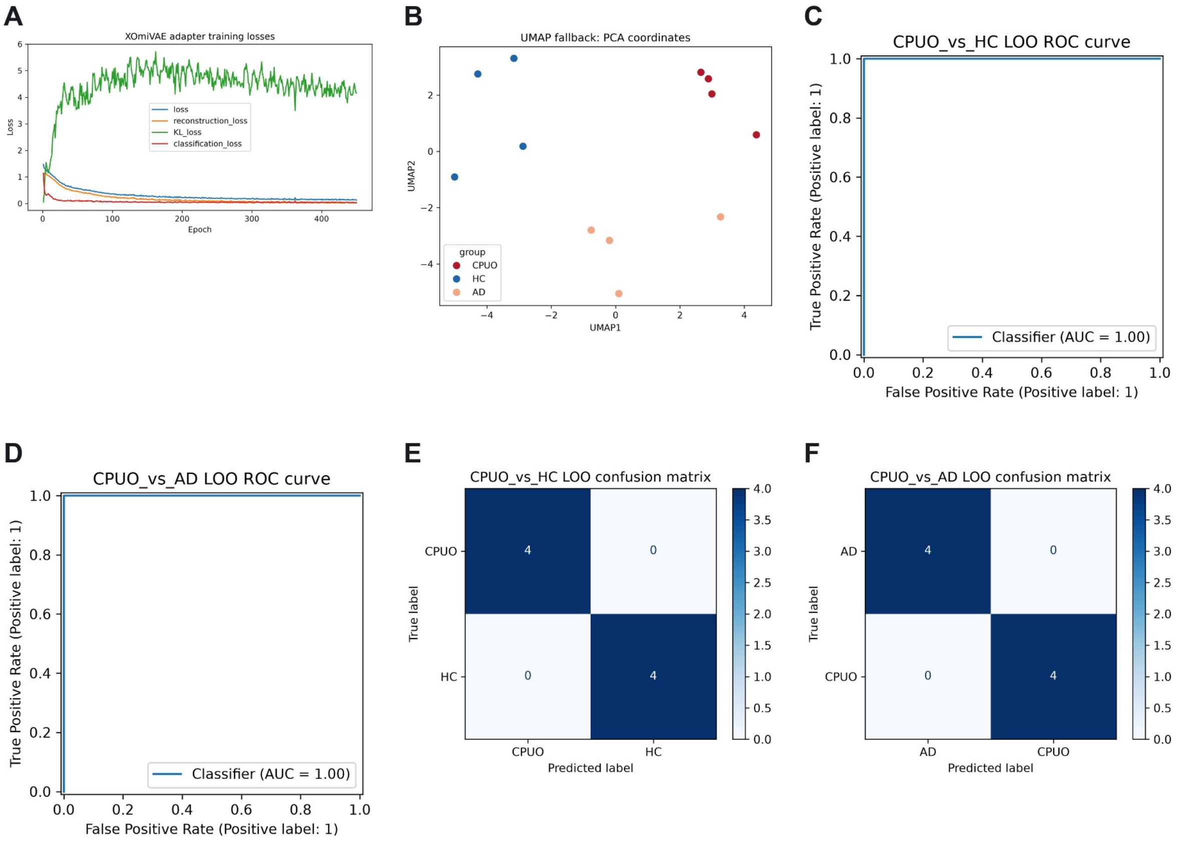

Exploratory performance only.

Supplementary Figure S4. Optional SHAP sensitivity analysis

(A) SHAP value bar plot for the surrogate classifiers. (B) SHAP summary plot. (C) Overlap between the SHAP-ranked genes of each surrogate model and the 201-gene signature at the top 10, 20, 50 and 100 cutoffs; the overlap is zero at every cutoff for all three model families. DeepSHAP could not be computed for the adapted model itself, so these values describe the surrogate classifiers only.

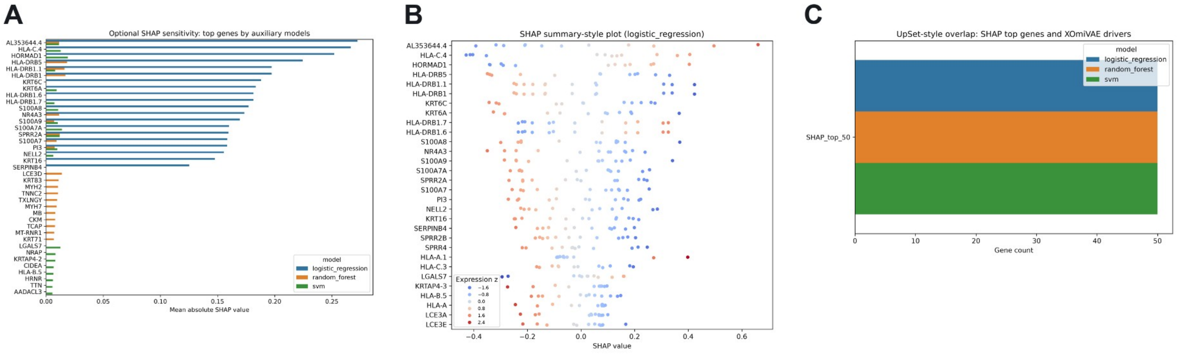

SHAP is optional sensitivity analysis; XOmiVAE remains the primary interpretability method.

Supplementary Figure S5. PBMC scRNA-seq QC, clustering, and markers

(A) Quality-control metrics for the GSE295457 PBMC dataset. (B) UMAP of Leiden clusters. (C) t-SNE of the same clusters. (D-F) Feature maps for the lineage markers CD3D (T cells), NKG7 (NK cells) and LST1 (myeloid cells) used to support the marker-based annotation reported in the main text.

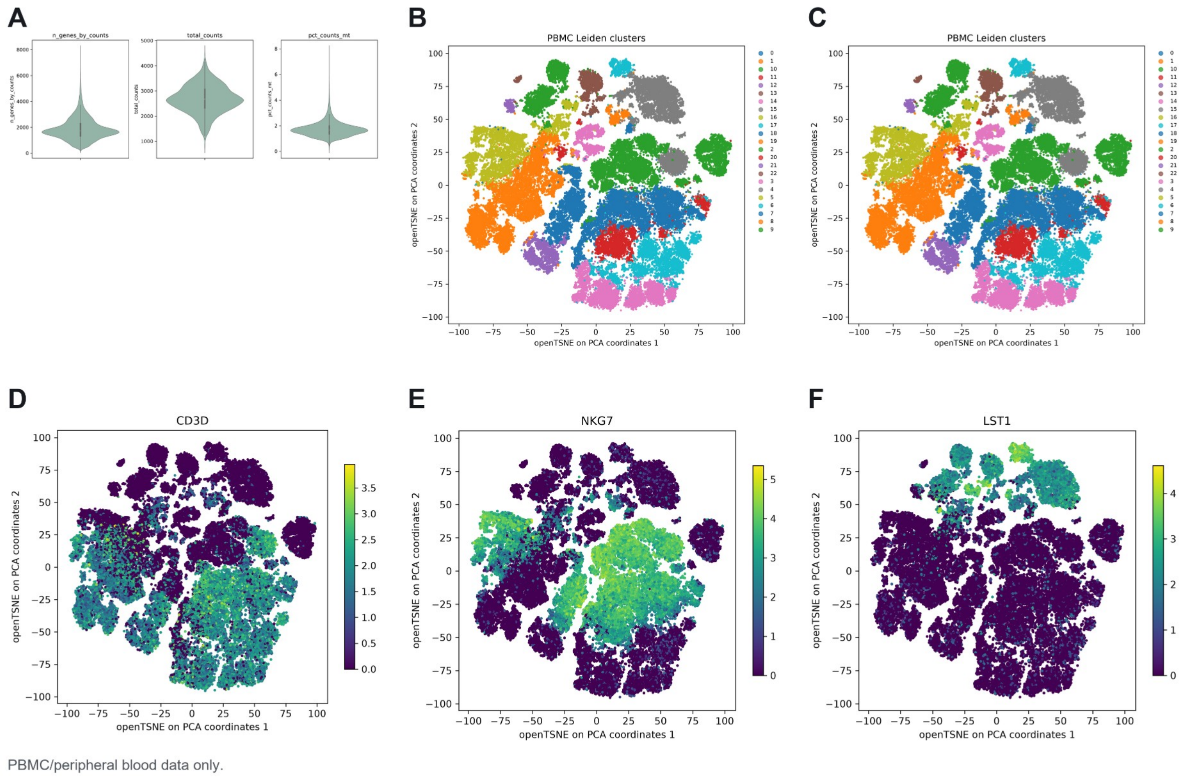

Supplementary Figure S6. Additional PBMC signature module maps

(A) Overall CPUO latent signature score projected onto the PBMC UMAP. (B-E) Module-level scores for the neuro-sensory, myeloid-immune, stromal-vascular and keratinocyte-stress programs. These maps show where skin-derived module genes are expressed among circulating cells and are descriptive; they do not test for differences between CPUO and healthy control.

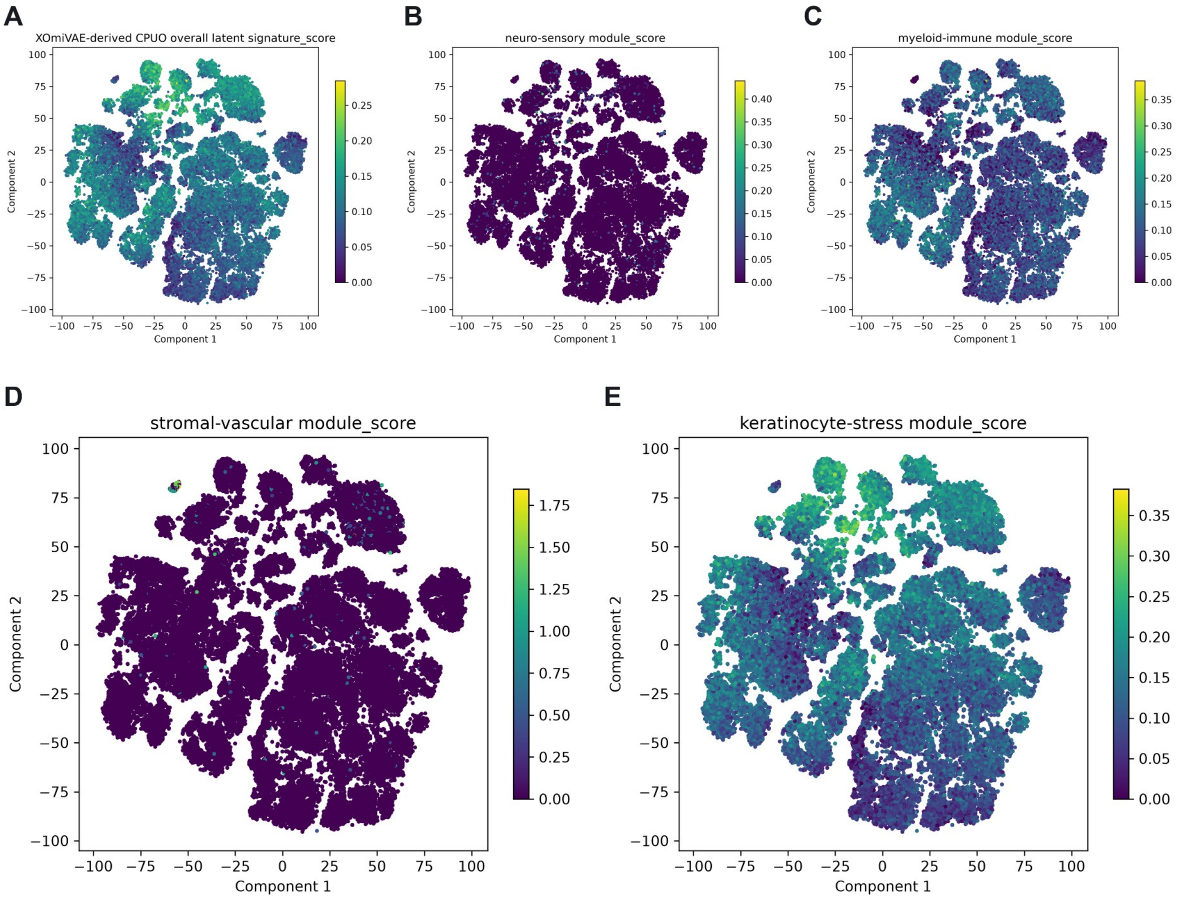

Supplementary Figure S7. Additional public skin spatial reference panels

(A) Neuro-sensory module score across the public skin spatial reference (GSE202011). (B) Radar plot summarizing module scores by annotated spatial neighborhood. The reference is a psoriatic-disease dataset, so region labels describe psoriatic rather than CPUO skin.

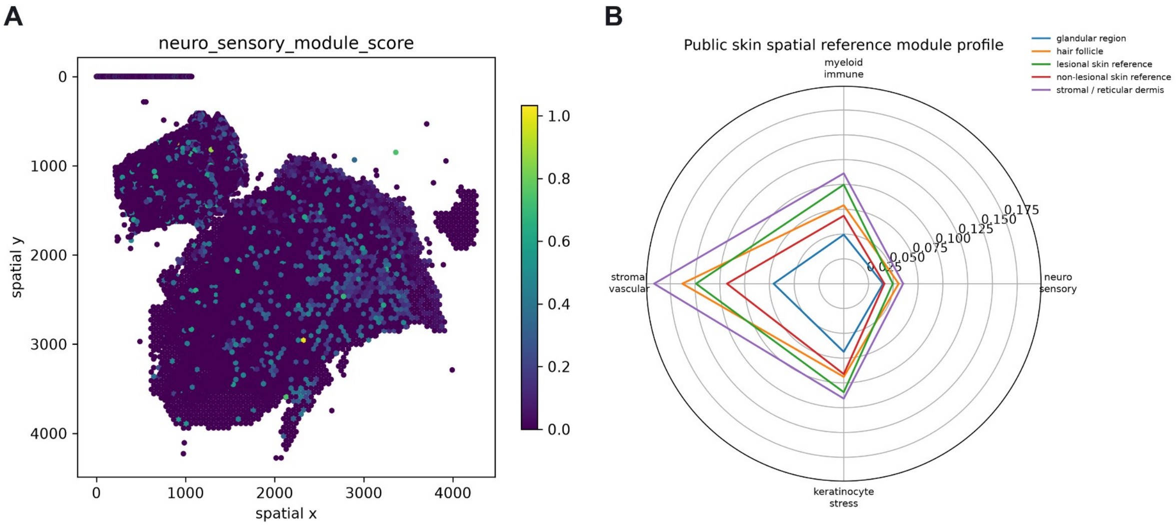

Public skin reference atlas only.

Supplementary Figure S8. Additional fibroblast-myeloid reference evidence

(A) Region-level dot plot of the candidate fibroblast-myeloid ligand-receptor pairs in the public skin spatial reference (GSE202011), providing anatomical context for their co-detectability in inflamed skin. This is atlas-guided context, not evidence of ligand-receptor activity in CPUO.

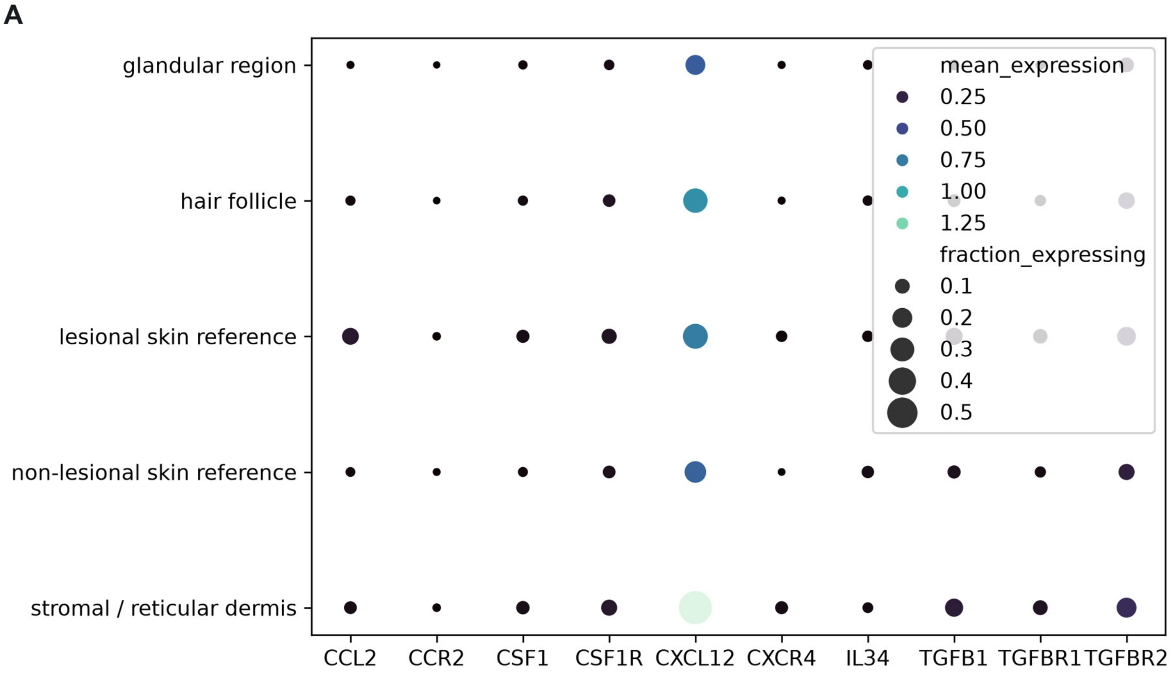

Supplementary Figure S9. Additional DRG atlas-guided interpretation

(A) Dot plot of canonical itch- and sensory-neuron-related genes across standardized cell types of the public human DRG atlas (GSE245310). (B) Dot plot of the candidate skin-to-DRG ligand-receptor pairs across the same cell types. The atlas profiles human embryonic DRG (gestational weeks 7-21), so these distributions describe developing ganglia and are not CPUO patient measurements.

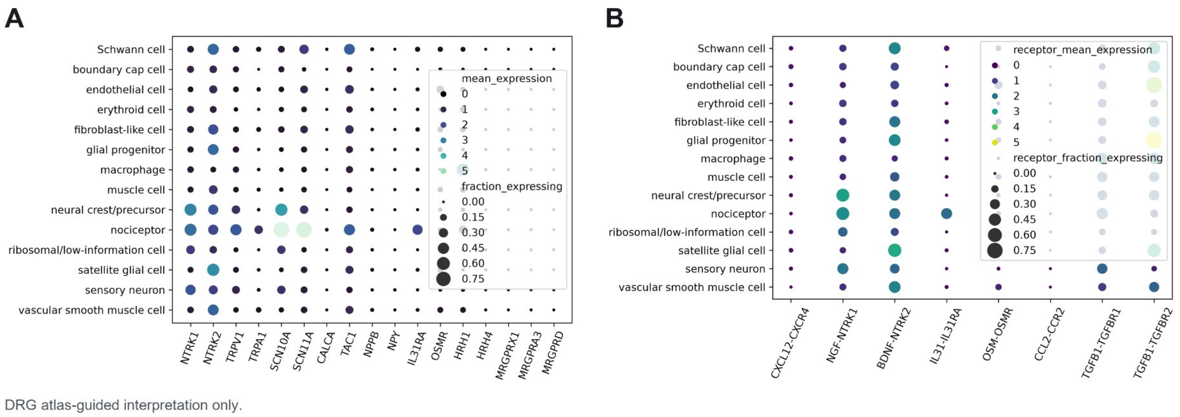

Supplementary Figure S10. CPUO niche score validation panels

(A) Exploratory receiver operating characteristic curves for the composite niche score (CPUO versus HC and CPUO versus AD), reported for completeness only: component orientation was fixed within the same 12 samples, so these curves are circular and internal to the discovery cohort. (B) Held-out composite scores from leave-one-out evaluation in which both z-scoring and component sign alignment were re-derived from the 11 training samples of each fold, so that the held-out sample contributed to neither. (C) Null distribution of the CPUO-versus-rest AUC across 5,000 label permutations, with the full orientation-and-scoring procedure repeated inside each permutation; the observed value is exceeded in 0.66% of permutations.

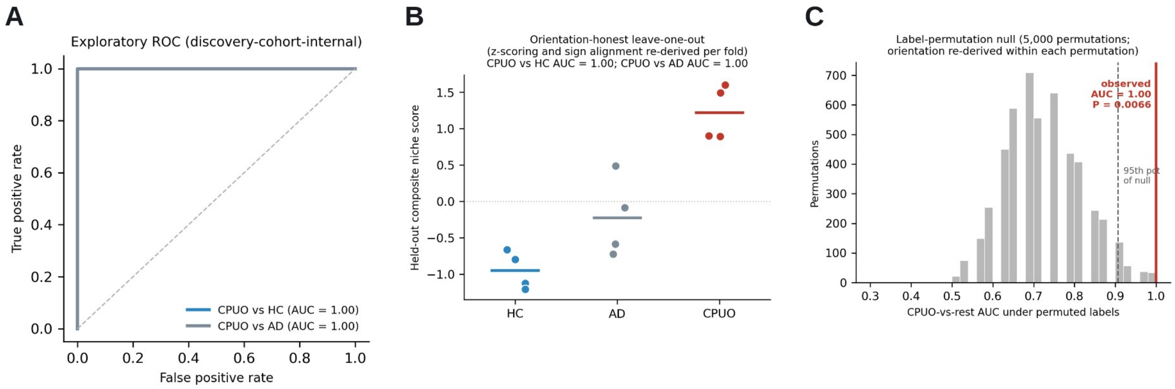

ROC is exploratory and internal to the discovery cohort; it is reported for completeness only. Panels B and C bound how much of the observed separation is attributable to within-cohort orientation of the score components.

Supplementary Table S1. Sample-level metadata for the analysed public series. All fields deposited for GSE237920 (skin) and GSE295457 (PBMC) are listed for every sample, together with an explicit record of the clinical variables that are not deposited - age, sex, disease duration, itch severity, treatment history and biopsy site - none of which could therefore be modeled or adjusted for in this study.

Supplementary Table S2. Complete 201-gene CPUO-driving signature. Gene symbol, module assignment, module assignment basis (keyword rule versus expression-correlation fallback), assignment confidence, gene-level contribution score, CPUO-driving score, evidence-category count and current gene-type annotation for every gene in the signature. Gene types were matched by exact symbol using Bioconductor org.Hs.eg.db version 3.20.0 (accessed 10 September 2026); unresolved legacy identifiers are explicitly retained as unresolved.

Supplementary Table S3. Cell-type-restricted PBMC pseudo-bulk differential expression (CPUO vs HC). Per-compartment results for classical monocytes, non-classical monocytes, monocytes combined, NK cells, T cells combined and dendritic cells, with the CCL3-CCR1 axis reported separately alongside the corresponding whole-cell estimates.

Supplementary Tables S1-S3 are provided in Supplementary_Tables_S1-S3.xlsx. The complete Table S3 results are included in worksheet S3e_celltype_DE_full; the README worksheet explains the contents, column definitions and data sources.
